# Modeling cardiac microcirculation for the simulation of coronary flow and 3D myocardial perfusion

**DOI:** 10.1007/s10237-024-01873-z

**Published:** 2024-07-12

**Authors:** Giovanni Montino Pelagi, Francesco Regazzoni, Jacques M. Huyghe, Andrea Baggiano, Marco Alì, Silvia Bertoluzza, Giovanni Valbusa, Gianluca Pontone, Christian Vergara

**Affiliations:** 1 LABS, Dipartimento di Chimica, Materiali e Ingegneria Chimica Giulio Natta, Politecnico di Milano, Piazza Leonardo da Vinci 32, Milan, 20133 Italy; 2 MOX, Dipartimento di Matematica, Politecnico di Milano, Piazza Leonardo da Vinci 32, Milan, 20133 Italy; 3 School of Engineering, University of Limerick, Limerick, V94 T9PX Ireland; 4 Eindhoven University of Technology, 5600 MB Eindhoven, The Netherlands; 5 Perioperative Cardiology and Cardiovascular Imaging Department, Centro Cardiologico Monzino IRCCS, Via Carlo Parea 4, Milan, 20138 Italy; 6 Department of Clinical Sciences and Community Health, University of Milan, Milan, Italy; 7 Bracco Imaging S.p.A., Via Caduti di Marcinelle 13, Milan, 20134 Italy; 8 Department of Diagnostic Imaging and Stereotactic Radiosurgery, Centro Diagnostico Italiano S.p.A., Via Saint Bon 20, Milan, 20147 Italy; 9 IMATI, CNR, Pavia, Italy; 10 Department of Biomedical, Surgical and Dental Sciences, University of Milan, Milan, 20134 Italy

**Keywords:** Coronary artery disease, Fractional flow reserve, Myocardial perfusion, Myocardial blood flow, Computational modeling, Coronary pressure

## Abstract

Accurate modeling of blood dynamics in the coronary microcirculation is a crucial step toward the clinical application of in silico methods for the diagnosis of coronary artery disease. In this work, we present a new mathematical model of microcirculatory hemodynamics accounting for microvasculature compliance and cardiac contraction; we also present its application to a full simulation of hyperemic coronary blood flow and 3D myocardial perfusion in real clinical cases. Microvasculature hemodynamics is modeled with a *compliant* multi-compartment Darcy formulation, with the new compliance terms depending on the local intramyocardial pressure generated by cardiac contraction. Nonlinear analytical relationships for vessels distensibility are included based on experimental data, and all the parameters of the model are reformulated based on histologically relevant quantities, allowing a deeper model personalization. Phasic flow patterns of high arterial inflow in diastole and venous outflow in systole are obtained, with flow waveforms morphology and pressure distribution along the microcirculation reproduced in accordance with experimental and in vivo measures. Phasic diameter change for arterioles and capillaries is also obtained with relevant differences depending on the depth location. Coronary blood dynamics exhibits a disturbed flow at the systolic onset, while the obtained 3D perfusion maps reproduce the systolic impediment effect and show relevant regional and transmural heterogeneities in myocardial blood flow (MBF). The proposed model successfully reproduces microvasculature hemodynamics over the whole heartbeat and along the entire intramural vessels. Quantification of phasic flow patterns, diameter changes, regional and transmural heterogeneities in MBF represent key steps ahead in the direction of the predictive simulation of cardiac perfusion.

## Introduction

Cardiac blood perfusion is the central physiological process that guarantees the metabolic sustenance of the heart muscle, requiring a dedicated circulatory system known as the coronary circulation. Defective perfusion is generally caused by a narrowing or blockage of a coronary artery, a condition known as coronary artery disease (CAD), and leads to major consequences such as myocardial ischemia, infarction and heart failure. Large clinical studies have shown that the combined knowledge of pressure drop in the large coronaries and myocardial blood flow (MBF) at the tissue level leads to the best management of patients suffering from CAD (Pontone et al. 2019; Baggiano et al. 2020). At present, however, this knowledge can be achieved only through multiple imaging examinations, often including radiation exposure and potentially invasive procedures (Knuuti et al. 2019).

In this context, mathematical models and computational simulations of coronary hemodynamics integrating blood flow in the large coronary arteries and myocardial perfusion hold great potential to provide clinically relevant information, especially when tailored to a specific subject using in vivo radiological images. Still, an accurate mathematical description of coronary hemodynamics remains a challenge because of two main reasons: Firstly, the coronary circulation spans over a broad range of length scales (from few millimeters to few microns of vessel diameter), making it impossible to run even 1D fluid dynamics simulations in the fully resolved tree; secondly, cardiac contraction deeply affects coronary flow, mainly through the well known systolic impediment effect (Chilian and Marcus 1982), which is challenging to model in an effective way. To address the first issue, previous works have proposed either a focus on large coronaries with outflow conditions, surrogating microvasculature, based on lumped parameter models (Olufsen et al. 2000; Anselmi et al. 2021) or on extended Murray’s law (Guerciotti et al. 2017); or multiscale models, often treating blood dynamics in the microcirculation through a homogenized porous medium approach [Darcy equations, Michler et al. (2013)], coupled with a 1D (Papamanolis et al. 2021) or 3D (Zingaro et al. 2023) description of fluid dynamics in the large coronaries. This has been further extended with the proposal of multi-compartment Darcy formulations to account for the different length scales in the microcirculation (Huyghe et al. 1989, 1989; Gregorio et al. 2021; Di Gregorio et al. 2022).

To cope with cardiac contraction, previous approaches relied on poromechanics as a way to model flow through a saturated porous medium subjected to mechanical activation (Huyghe et al. 1992; Vankan et al. 1997; Chapelle et al. 2009), possibly coupled with coronary arterial networks (Lee et al. 2015; Richardson et al. 2021; Barnafi Wittwer et al. 2022). In a previous work (Pelagi et al. 2024), we proposed an *effective* inlet pressure condition for the large coronaries to surrogate the effects of cardiac contraction in a multiscale coupled model of cardiac perfusion. However, the first models are difficult to personalize and have never been applied to real clinical scenarios, whereas what proposed in Pelagi et al. (2024) does not provide a sufficient accuracy for the distribution of blood flow at the tissue level.

In this work, to overcome these limitations, we start from the multiscale perfusion model presented in Gregorio et al. (2021) and we propose:

1. A new mathematical formulation of the multi-compartment Darcy model to account for cardiac mechanics and microvasculature compliance;
2. A new, data-driven calibration of the Darcy model parameters in view of simulations of hyperemic coronary flow in real clinical cases.

This new model for microvascular hemodynamics is coupled with 3D fluid dynamics equations for the blood dynamics in the large coronaries to run an integrated analysis (at all levels of the coronary tree) in subjects whose heart and coronary geometries have been reconstructed from in vivo CT images.

To the best of our knowledge, this is the first computational model, incorporating details along all the coronary tree and effects of cardiac contraction on perfusion, which has been calibrated for an application to real clinical cases. We believe that this work is a crucial step toward a predictive application of perfusion modeling in a clinical setting and the use of computational methods to reproduce functional imaging of stress computed tomographic perfusion (stress-CTP) (Pontone et al. 2019).

## Methods

In Sect. 2.1, we introduce the new mathematical formulation for the multi-compartment Darcy model in a domain subjected to a cyclic mechanical stress caused by cardiac contraction. In Sects. 2.2 and 2.3, we provide an overview of the new parameters introduced together with our choices leading to the surrogating contraction model and we propose a data-driven approach for their calibration, whereas in Sect. 2.4, we detail how we model the intramyocardial pressure. In Sect. 2.5 we detail the coupling of the proposed multi-compartment Darcy model with the hemodynamics in the epicardial coronaries. Section 2.6 includes the details regarding the time discretization and linearization of the proposed model, while in Sect. 2.7 we describe how we generate the computational domain as well as the general setup used for the numerical simulations, together with the fixed-point strategy used to manage the coupled problem.

### Model of microcirculation hemodynamics

When the coronary arteries penetrate the myocardial surface, they progressively branch into smaller vessels in a tree-like structure known as the *intramural* circulation. Given the huge number of vessels, a homogenized approach where hemodynamics is described as a flow through a porous medium is well suited to describe hemodynamics in this part of the coronary tree (Huyghe et al. 1992; Vankan et al. 1996; Michler et al. 2013).

**Fig. 1:**
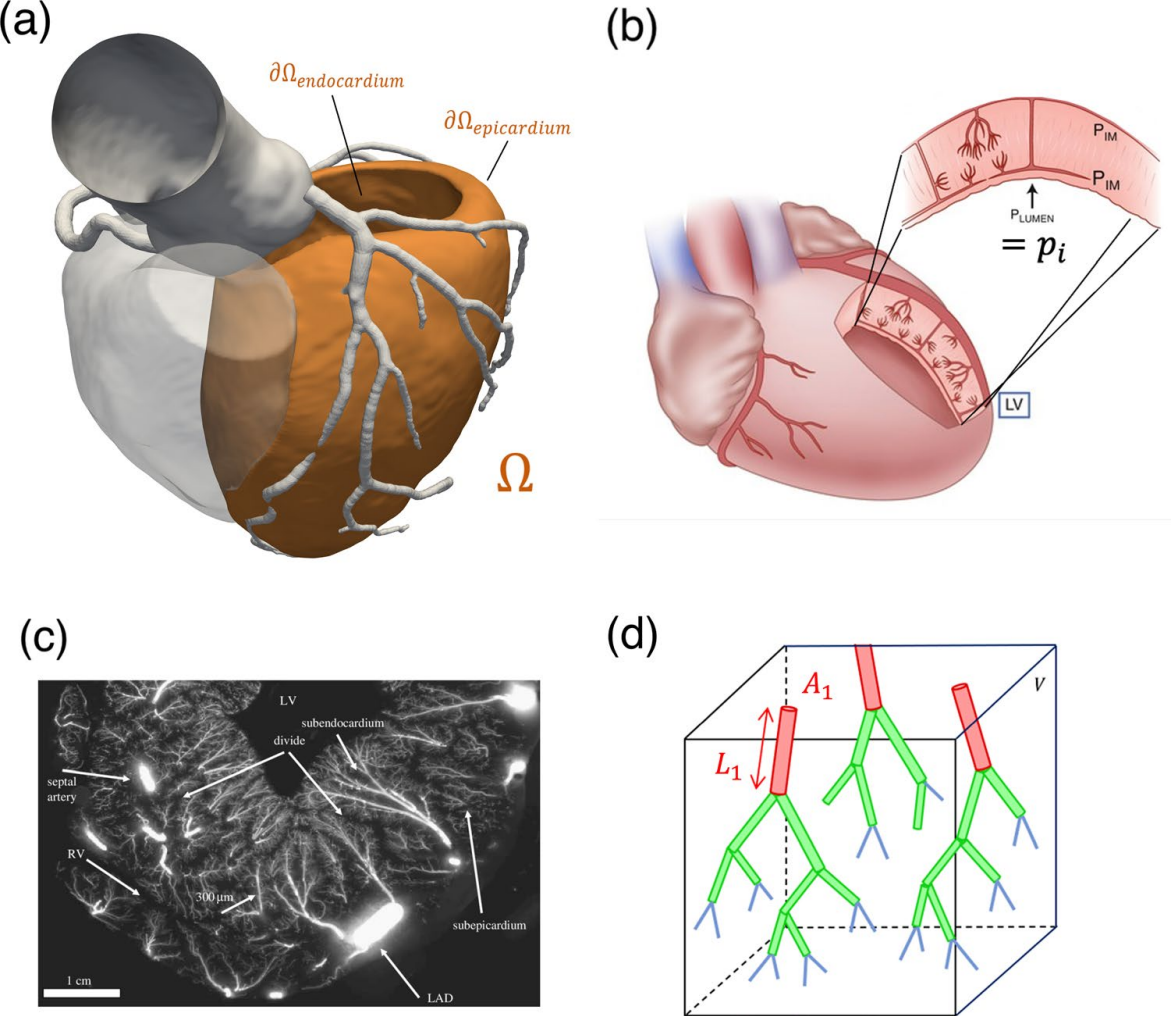
**a** Computational domain $\Omega$ (consisting of the left ventricular free wall, in orange) for the microcirculation model. Aortic root, epicardial coronary tree and right ventricular chamber (shaded) are also displayed but not a part of $\Omega$. **b** Schematic representation of the coexistence of intramural vessels and myocardial tissue within the left ventricular free wall (adapted from Duncker and Bache (2008)). **c** Ex vivo cryomicrotome image of coronary intramural circulation showing the organization of vessels within the ventricular wall (reproduced from Spaan et al. (2008)). **d** Schematic visualization of the hierarchical organization of the intramural network within a homogenization volume *V*: coloring represents the belonging of a vessel to a specific class (i.e., small arteries, arterioles or capillaries) which corresponds to a specific Darcy compartment in our model. Vessels cross section $A_i$ and average length $L_i$ are indicated for the first compartment, with vessels density $n_i = \frac{N_i}{V}$ for each compartment *i*

To account for the different length scales (diameters from $d \simeq {5\,\mathrm{\upmu \text {m}}}$, capillaries, up to $d \simeq {500\,\mathrm{\upmu \text {m}}}$, small arteries) as well as for the mechanical activity of the heart, we start from the three-compartment primal Darcy formulation presented in Gregorio et al. (2021), Michler et al. (2013) and we generalize it with the addition of a compliance term resulting from vessels distensibility. For the compartments, we consider the following subdivision: small arteries (comp. 1, *d* between 100 and 500 ${\,\mathrm{\upmu \text {m}}}$), arterioles (comp. 2, *d* between 8 and 100 ${\,\mathrm{\upmu \text {m}}}$), capillaries (comp. 3, *d* between 4 and 8 ${\,\mathrm{\upmu \text {m}}}$). The strong formulation for a generic compartment $i = 1,2,3$ reads:

1 $$\begin{aligned} {\left\{ \begin{array}{ll} \displaystyle - \nabla \cdot (K_i \nabla p_i) + \frac{\partial \phi _i}{\partial t} + \sum _{j=1}^{3} \beta _{i,j}(p_i - p_j) = \theta _i &{} \text { in }\, \Omega , \\ \phi _i = f_i(p_i, P_{\textrm{im}}) &{} \text { in }\, \Omega , \\ &{} \\ \displaystyle \frac{\partial p_i}{\partial {\varvec{n}}} = 0 &{} \text { on }\, \partial \Omega , \\ \end{array}\right. } \end{aligned}$$

where, for each compartment *i*, $p_i$ is the unknown intraluminal blood pressure, $K_i$ is the permeability (considered as a scalar field), $\beta _{i,j}$ is the mass exchange coefficient with compartment *j*; $\theta _i$ is a distributed mass source/sink term accounting both for the mass source in compartment 1 that represents the flow coming from the large arteries (for example provided by the solution of a Navier–Stokes problem, see Gregorio et al. (2021); Di Gregorio et al. (2022) and Sect. 2.7) and for the mass sink in compartment 3 representing the venous return, i.e., $\theta _3 = -\gamma (p_3 - p_{\text{ra}})$, $p_{\text{ra}}$ being the right atrium pressure. Notice that the equations related to each compartment are solved in the same computational domain $\Omega$, that is the left ventricular free wall reported in Fig. 1a, meaning that we assume each compartment of intramural vessels to coexist in the same volume.

The new compliance term $\frac{\partial \phi _i}{\partial t}$ represents the time variation of the fluid volume fraction $\phi _i$ (i.e., the porosity of the $i$th compartment), which we model with a suitable set of functions $f_i$ representing the relationship between compartment porosity, the intraluminal pressure $p_i$ and the given intramyocardial pressure $P_\textrm{im}$, generated within the cardiac tissue by the heart contraction. A schematic representation of the intraluminal/extraluminal spaces with their pressures is reported in Fig. 1b, while in Sect. 2.4 we propose a specific treatment for the computation of $P_\textrm{im}$, with the main modeling assumption that $P_\textrm{im}$ is considered independent of the intraluminal blood pressure and prescribed as a given datum.

Considering the Darcy compartments as networks of cylindrical vessels (as schematized in Fig. 1d), the local fluid volume fraction of compartment *i* in a given homogenization volume *V* can be written, by definition, as:

$$\begin{aligned} \phi _i = \frac{V_{f,i}}{V} = \frac{N_i L_i A_i}{V} = (nL)_i A_i, \end{aligned}$$

where $N_i$ is the total number of vessels belonging to compartment *i*, $L_i$ is the average vessel length and $A_i$ is the average vessel cross section. The ratio $n_i = \frac{N_i}{V}$ is the local density of vessels belonging to compartment *i*, whereas the product $(nL)_i$ is often denoted as the vessel *length density*. Given that the intramural vessels are modeled as cylinders with a relatively thin wall, we assume that cardiac contraction affects exclusively the cross-sectional area, whereas the vessels density and length remain constant. Further, we assume that vessels cross section explicitly depend on the transluminal pressure difference $(p_i - P_\textrm{im})$, as supported by experimental measures (Spaan 1985). Under these hypotheses, the time derivative of the fluid volume fraction becomes:

2 $$\begin{aligned} \displaystyle \frac{\partial \phi _i}{\partial t} = (nL)_i \frac{\partial A_i}{\partial (p_i - P_\textrm{im})} \frac{\partial (p_i - P_\textrm{im})}{\partial t} = (nL)_i C_i \frac{\partial (p_i - P_\textrm{im})}{\partial t}, \end{aligned}$$

where

3 $$\begin{aligned} C_i = \frac{\partial A_i}{\partial (p_i - P_\textrm{im})} \end{aligned}$$

represents the *distensibility* of vessels in compartment *i*, which is, in general, dependent on the transluminal pressure difference $(p_i - P_\textrm{im})$.

By substituting eq. (2) in (1), we obtain the final formulation of the *compliant* multi-compartment Darcy model:

4 $$\begin{aligned} {\left\{ \begin{array}{ll} \displaystyle - \nabla \cdot (K_i \nabla p_i) + (nL)_i C_i \frac{\partial (p_i - P_\textrm{im})}{\partial t} + \sum _{j=1}^{3} \beta _{i,j}(p_i - p_j) = \theta _i &{} \quad \text { in } \Omega , \\ \displaystyle \frac{\partial p_i}{\partial {\varvec{n}}} = 0 &{} \quad \text {on } \partial \Omega . \\ \end{array}\right. } \end{aligned}$$

for $i = 1,2,3$. Notice that parameters $K_i$, $\beta _{i,j}$ and $C_i$ in (4) are dependent on the transluminal pressure difference $(p_i - P_\textrm{im})$ and are computed using the methods exposed in the following two sections.

### Constitutive relations for vessel distensibility

As seen by its definition (3), vessels distensibility $C_i$ represents the variation of cross-sectional area with respect to variations in transluminal pressure difference. Since the histological structure of coronary microvessels changes depending on their diameter, the constitutive relationships we used for $C_i$ are compartment-specific and their choice is driven by experimental data. It is important to note that, since we are interested in hyperemic coronary flow, we used data related only to a vessel condition of maximal vasodilation, ruling out the effects of vascular tone and autoregulation mechanisms. This is motivated by the fact that, at maximal hyperemia, these mechanisms are exhausted and the vessel wall can be modeled as a fully passive structure.

**Fig. 2:**
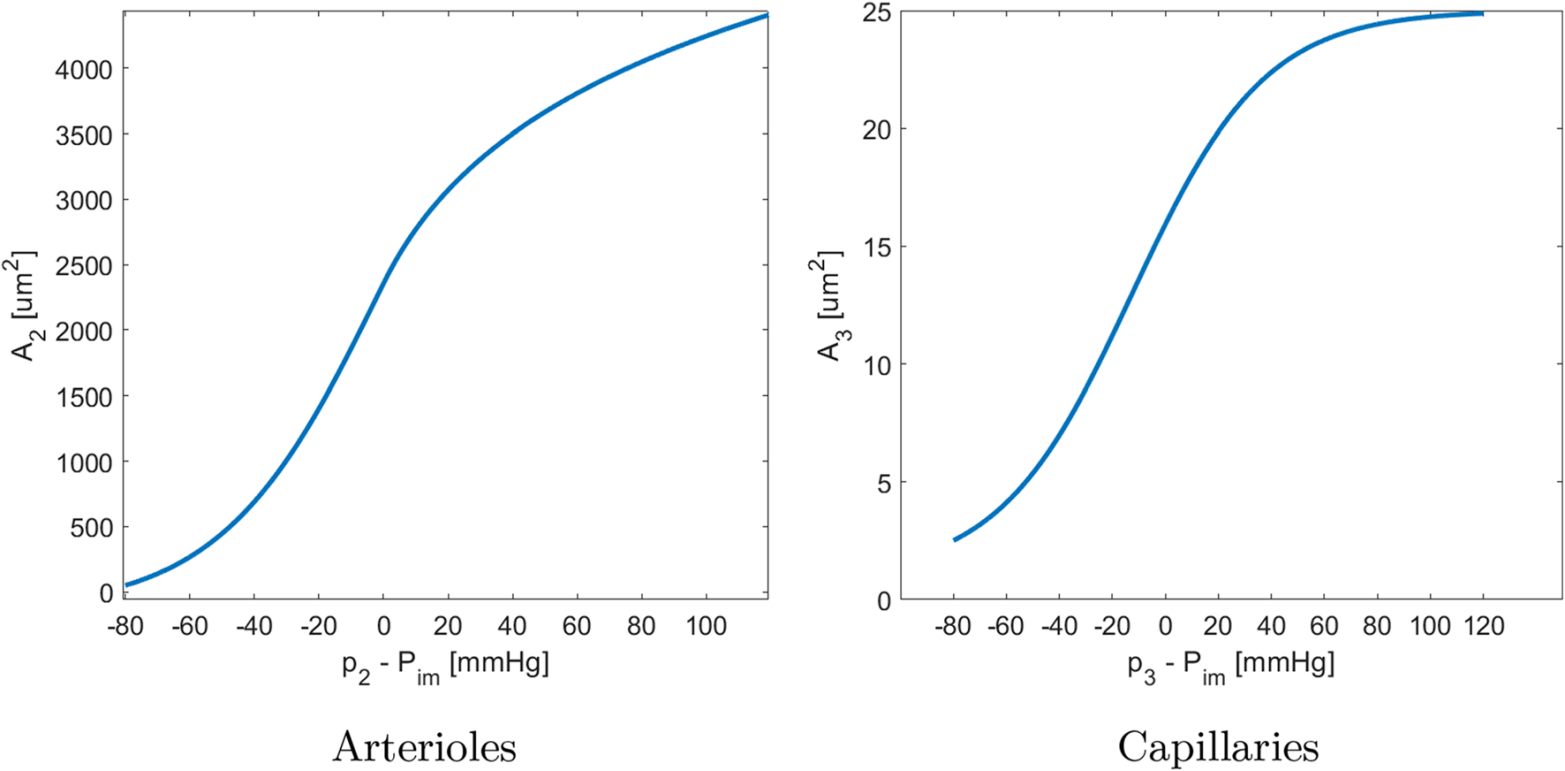
Constitutive curves relating vessels cross section and transluminal pressure for arterioles and capillaries

For the compartment-specific constitutive relationships we consider what follows:

1. Small arteries (comp. 1) have a relatively thick wall structure consisting of collagen and smooth muscle cells, and thus we consider these vessels as rigid and we set
  5 $$\begin{aligned} A_1 [{\,\mathrm{mm^2}}] = 0.07, \end{aligned}$$
  $$\begin{aligned} C_1 = 0; \end{aligned}$$
  where the specific value chosen for $A_1$ corresponds to a diameter $d = 150$ μm, which we consider as mean diameter for vessels of this class.
2. Arterioles (comp. 2) have been found to be much more distensible than the small arteries during in vivo observations in animal experiments (Hiramatsu et al. 1998; Yada et al. 1993). Also, experimental measures of arterioles have shown a highly nonlinear relationship between transluminal pressure and cross-sectional area (Spaan 1985). To capture this behavior, we fitted an analytical relationship on data from isolated arterioles (Spaan 1985) (see Fig. 2); a logarithmic expression is chosen as best fit of the data. Since we have at disposal data only for $p_2 - P_{\textrm{im}} > 0$, we extend this curve through a sigmoid function in the negative region. The analytical expression is given by:
  6 $$\begin{aligned} A_2 \, [{\,\mathrm{mm^2}}] = {\left\{ \begin{array}{ll} \displaystyle 0.0030 + \frac{0.0050}{(1 + e^{-3.02*10^{-4}(p_2-P_{\textrm{im}})})} \qquad &{}\text {if } \quad p_2 - P_{\textrm{im}} < 0; \\ 0.0011 \ln [1.2(p_2-P_{\textrm{im}}) + 3500] -0.0033 \qquad &{}\text {if } \quad p_2 - P_{\textrm{im}} \ge 0; \end{array}\right. } \end{aligned}$$
  Coefficients for the logarithmic fit in the positive region of (6) are computed with a least-square method applied to data reported in Spaan (1985). Regarding the sigmoidal extension in the negative region, we assume an asymptotic minimum value of $A_2 = {0.003\,\mathrm{mm^2}}$, whereas the remaining three coefficients are computed to ensure continuity and continuity of first and second derivatives at the junction point ($p_2 - P_{\textrm{im}} = 0$). From (3), we obtain the expression for the arteriolar compliance $C_2$:
  $$\begin{aligned} C_2 \, \left[ {\,\mathrm{mmHg \cdot Pa^{-1}}} \right] = {\left\{ \begin{array}{ll} \displaystyle \frac{1.51*10^{-6} e^{-3.02*10^{-4}(p_2-P_{\textrm{im}})}}{(e^{-3.02*10^{-4}(p_2-P_{\textrm{im}})}+1)^2} \qquad &{}\text {if } \quad p_2 - P_{\textrm{im}} < 0; \\ \displaystyle \frac{0.0013}{1.2(p_2-P_{\textrm{im}}) + 3500} \qquad &{}\text {if } \quad p_2 - P_{\textrm{im}} \ge 0. \end{array}\right. } \end{aligned}$$
3. Capillaries (comp. 3) do not have any muscle cells, having only a single layer of endothelial cells. However, experimental observations have shown that they are surprisingly resistant to systolic compression, exhibiting a relatively low change in diameter between the diastolic ($d \simeq {5.4\,\mathrm{\upmu \text {m}}}$) and systolic ($d \simeq {4.3\,\mathrm{\upmu \text {m}}}$) phases (Toyota et al. 2002). Differently from the case of arterioles, there are no experimental data relating capillary cross section and transluminal pressure. We assume as a modeling abstraction a curve for the capillary cross section built from the values of systolic/diastolic diameter in Toyota et al. (2002) with a sigmoid shape similar to $A_2$ (see Fig. 2b). Its analytical expression is given by:
  7 $$\begin{aligned} A_3 \, [{\,\mathrm{mm^2}}] = 1*10^{-6} + \frac{2.4*10^{-5}}{(1 + e^{-3*10^{-4}(p_3-P_{\textrm{im}}) -0.5})}, \end{aligned}$$
  Due to the low amount of data, coefficients in (7) are difficult to determine. Since the only available experimental observations are related to the systolic/diastolic diameters, we perform a first estimate by guessing the transluminal pressure difference across capillaries in the two phases and assuming an asymptotic minimum value of $A_3 = {1\,\mathrm{\upmu \text {m}^2}}$. Coefficients are later fine tuned through a trials-and-errors approach to obtain the most physiological results. From (3), we obtain the expression for the capillary compliance $C_3$:
  $$\begin{aligned} C_3 \, \left[ {\,\mathrm{mmHg \cdot Pa^{-1}}} \right] = \frac{7.2*10^{-9} e^{-3*10^{-4}(p_3-P_{\textrm{im}})-0.5}}{(e^{-3*10^{-4}(p_3-P_{\textrm{im}})-0.5}+1)^2}. \end{aligned}$$

### Estimation of Darcy parameters

For permeabilities $K_i$, we assume a direct proportionality with the porosity, which coincides with the fluid volume fraction (the porous medium is saturated):

8 $$\begin{aligned} K_i = \frac{\kappa }{\mu } \phi _i = \frac{\kappa }{\mu } (nL)_i A_i, \end{aligned}$$

where $\kappa$ is the specific permeability of the fluid/matrix system, considered as independent of the Darcy compartment, and $\mu$ is the blood dynamic viscosity; see the Discussion section for such choices.

The conductance coefficients $\beta _{i,j}$ mediate mass transfer between compartments, so for their formulation we start considering each vessel as a Poiseuille-like resistor with conductance (PFITZNER 1976):

9 $$\begin{aligned} \beta _{vessel,i} = \frac{A_i^2}{8 \pi \mu L_i}; \end{aligned}$$

then we extend this formula to the whole compartment, by considering it as a network of conductances in series and parallel, which is motivated by the tree-like structure of vessels networks. The conductance of the whole network is proportional to the one of the single vessels and to the vessels density $n_i$:

$$\begin{aligned} \beta _i \propto n_i \beta _{vessel,i}. \end{aligned}$$

Thus, exploiting (9), we obtain:

10 $$\begin{aligned} \beta _i = \delta ^*_i n_i \frac{A_i^2}{8 \pi \mu L_i} = \delta _i (nL)_i A_i^2, \end{aligned}$$

where $\delta ^*_i$ is a coefficient to account for the specific morphometry of the network. While length densities $(nL)_i$ are just a measure of the total length of vessels belonging to a certain class (normalized by volume), morphometry factors measure how these vessels are hierarchically arranged in space, incorporating information such as bifurcation/trifurcation ratio, fraction of vessel segments connected in series, and branching asymmetry in radii. While, in principle, these information can be extracted from detailed topological data (Kassab et al. 1993; Schwarz et al. 2020), such operation would not be straightforward, also requiring a clustering of vessels to discrete compartments. For this reason, we compute these parameters through a calibration procedure exposed in Sect. 2.7. To reduce the number of parameters, we group the geometry-related parameter $L_i$ and the viscosity $\mu$ into the final morphometry factor $\delta _i = \frac{\delta _i^*}{8 \pi \mu L^2_i}$. This formulation allows us to have an explicit dependency of the conductance coefficients $\beta _{i,j}$ on the length densities $(nL)_i$, which are the most used parameters to describe the degree of vascularization and can, in principle, be space-dependent.

Since we can assume that mass exchanges between compartments depend on the conductances of both the upstream and downstream networks, we finally take as inter-compartment conductance $\beta _{i,j}$ the expression:

11 $$\begin{aligned} \beta _{i,j} = \frac{\beta _i + \beta _j}{2} = \frac{(nL)_i \delta _i A_i^2 + (nL)_j \delta _j A_j^2}{2}. \end{aligned}$$

Parameters $\kappa$ in eq. (8) and $\delta _i$ in eq. (11) are difficult to estimate from data, so for their computation we rely on a calibration procedure exposed in Sect. 2.7, alongside a list of all the other parameters used in the simulations (see Table 1).

### Estimation of the intramyocardial pressure

The knowledge of the intramyocardial pressure $P_{\textrm{im}}$ is fundamental to build curves $A_i$ and $C_i$ as well as the compliance term $\frac{\partial \phi _i}{\partial t}$ in (1). It allows to include the effect of cardiac mechanics on microcirculation hemodynamics. According to previous studies (Algranati et al. 2010), most of the experimental observations related to coronary hemodynamics can be explained by considering cyclic changes in $P_{\textrm{im}}$ induced by and closely following the pressure $P_\textrm{LV}$ generated inside the ventricular chamber. Other mechanisms, such as the shortening-induced intracellular pressure, were found to play a role only in the case of specific states of altered contractility.

**Fig. 3:**
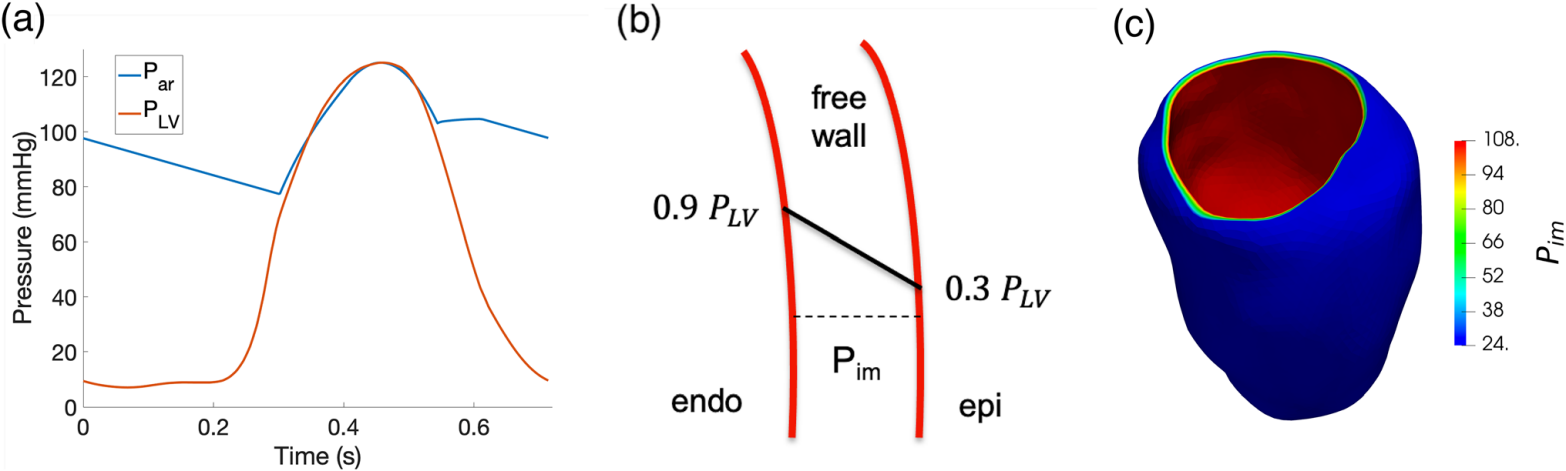
**a** Time waveform of the pressure in the left ventricular chamber, obtained with an electromechanics simulation (Fedele et al. 2023). **b** Transmural modulation of intramyocardial pressure $P_{\textrm{im}}$ in the left ventricular free wall. **c** 3D representation of the transmural modulation displayed at systolic peak

Given these findings, we consider for the temporal waveform of $P_{\textrm{im}}$ the left ventricular pressure $P_\textrm{LV}$, obtained from an electromechanics simulation (Fedele et al. 2023). Starting from the base waveform, we obtain personalized $P_\textrm{LV}$ curves by matching the systolic interval to the one of the patient and the peak $P_\textrm{LV}$ pressure to the peak of the patient’s aortic pressure. The personalized $P_\textrm{LV}$ curve is reported in Fig. 3a, which includes also a comparison with the aortic pressure.

Since experimental findings pointed out that intramyocardial pressure decreases almost linearly from the subendocardium to the subepicardium (Baird et al. 1970), we include a linear transmural modulation of $P_{\textrm{im}}$ such that:

12 $$\begin{aligned} {\left\{ \begin{array}{ll} P_{\textrm{im}} = 0.9 \, P_\textrm{LV} &{} \text { at } \partial \Omega _{endocardium}, \\ P_{\textrm{im}} = 0.3 \, P_\textrm{LV} &{} \text { at } \partial \Omega _{epicardium}, \\ \end{array}\right. } \end{aligned}$$

which is in accordance with the quantitative experimental data in Baird et al. (1970), Heineman and Grayson (1985). A representation of this transmural modulation is reported at the systolic peak in Fig. 3b, together with a left ventricular distribution (see Fig. 3c). The final $P_{\textrm{im}}$ imposed, therefore, has a time evolution that follows the one of the pressure in the left ventricular chamber $P_\textrm{LV}$ and it is modulated in space according to (12), decreasing linearly from the endocardium to the epicardium. This means also that each perfusion territory is subjected to the same intramyocardial pressure.

### Coupling with hemodynamics in epicardial coronaries

**Fig. 4:**
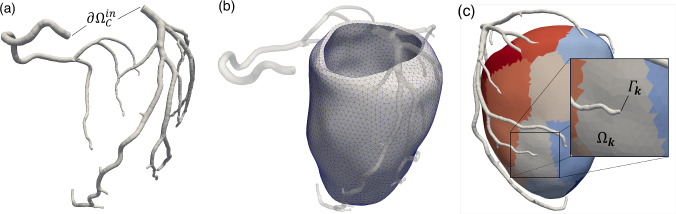
**a** Segmented domain used for the solution of the 3D hemodynamics problem. **b** Segmented domain, with mesh detail, used for the solution of the multi-compartment Darcy problem. **c** Representation of the coupling coupling between the two problems: Each coronary outlet $\Gamma _k$ is coupled with a corresponding perfusion territory $\Omega _k$

The new multi-compartment Darcy problem (4) with the parameters choices described in Sects. 2.2 and 2.3 is coupled with large epicardial coronaries hemodynamics, where a 3D fluid dynamics problem given by the incompressible Newtonian Navier-Stokes equations is considered (Gregorio et al. 2021) (see Fig. 4a). At the two coronary inlets $\partial \Omega _C^{in}$, we prescribe patient-specific pressure waveforms, that are built in a personalized way from patients’ measures of brachial pressure and heart rate, using the methodology we developed in Pelagi et al. (2024). Downstream, the epicardial coronaries are coupled with the Darcy model, representing microcirculation hemodynamics solved in the left ventricular free wall (Fig. 4b).

To couple the two subproblems, each coronary outlet $\Gamma ^k$ is associated with a perfusion territory $\Omega ^k$ in the myocardium (see Fig. 4c). Interface conditions representing force balance are prescribed at each coronary outlet based on the mean pressure of Darcy compartment 1 $p_1$ in the corresponding perfusion territory and on the coronary hemodynamic quantities $p_C$, ${\varvec{u}}_C$. As proposed in Gregorio et al. (2021), this results in the following defective boundary condition, completed with homogeneous tangential Neumann conditions for the NS problem:

13 $$\begin{aligned} \left\{ \begin{array}{ll} \displaystyle -p_C + \mu \left( \nabla {\varvec{u}}_C + (\nabla {\varvec{u}}_C)^T \right) {\varvec{n}} \cdot {\varvec{n}} = - \frac{1}{\vert \Omega ^k \vert } \int _{\Omega ^k} p_1 d{\varvec{x}} - \frac{1}{\alpha } \int _{\Gamma ^k} {\varvec{u}}_C \cdot {\varvec{n}} d \gamma &{} \quad \text {on } \Gamma ^k, \\ \displaystyle \mu \left( \nabla {\varvec{u}}_C + (\nabla {\varvec{u}}_C)^T \right) {\varvec{n}} \cdot \varvec{\tau }_i = 0 \quad i = 1, 2 &{} \quad \text {on } \Gamma ^k, \\ \end{array} \right. \end{aligned}$$

where ${\varvec{u}}_C$ is the coronary velocity unknown, $|\Omega ^k |$ is the volume of the perfusion territory $\Omega ^k$ and $\alpha$ is the coupling conductance, whose value is reported in Table 1.

Interface conditions representing mass conservation are prescribed in the Darcy problem through the following source terms $\theta _i$:

14 $$\begin{aligned} {\left\{ \begin{array}{ll} \displaystyle \theta _1 = \sum _{k=1}^N \frac{\chi ^k}{|\Omega ^k |} \int _{\Gamma ^k} {\varvec{u}}_C \cdot {\varvec{n}}, \text { with } \chi ^k({\varvec{x}}) = {\left\{ \begin{array}{ll} 1 \text { if } {\varvec{x}} \in \Omega ^k, \\ 0 \text { if } {\varvec{x}} \in \Omega ^k, \end{array}\right. } \\ \theta _2 = 0, \\ \theta _3 = -\gamma (p_3 - p_{\text{ra}}). \\ \end{array}\right. } \end{aligned}$$

In the expression for $\theta _1$ from (14), which represents the mass source in the first Darcy compartment, *N* is the total number of coronary outlets, the term $\int _{\Gamma ^k} {\varvec{u}}_C \cdot {\varvec{n}}$ is the flow through the coronary outlet $\Gamma ^k$ and $\chi ^k$ is a characteristic function ensuring that each perfusion territory receives blood only from the corresponding coronary outlet $\Gamma ^k$. Notice also the expression for $\theta _3$ (14), representing the mass sink in the third Darcy compartment due to the venous return and featuring the constant parameters $\gamma$ (conductance of the whole coronary venous circulation) and $p_{\text{ra}}$ (right atrium pressure), whose values are reported in Table 1.

Myocardial partitioning and the association between feeding arteries and a specific perfusion territory are performed with the following distance approach:

1. The barycenter of each coronary outlet $\Gamma ^k$ is projected onto the ventricular geometry. In this step, we find the points $Q^k$ in the myocardium that are closest to the corresponding coronary outlet;
2. For each projected outlet, we compute a distance $d^k$ through the solution of an eikonal problem in the ventricular geometry:
  15 $$\begin{aligned} {\left\{ \begin{array}{ll} \displaystyle \vert \nabla d^k \vert = \frac{1}{r^k} \quad &{} \text {in } \Omega , \\ \displaystyle d^k = 0 &{} \text {on } Q^k, \\ \end{array}\right. } \end{aligned}$$
  where $r^k$ is the radius of the coronary outlet $\Gamma ^k$. The distances $d^k$ found this way, therefore, represent modified distances with respect to the euclidean ones, to take into account the radius of the feeding arteries;
3. Myocardial partitioning is performed exploiting the Voronoi tessellation algorithm, assigning each point in the myocardium to a specific perfusion region $\Omega _k$ (so the corresponding outlet points $Q^k$) if the modified distance $d^k$ is the minimum across all the modified distances computed this way.

In comparison with the approach based on euclidean distance proposed in Gregorio et al. (2021), the previous procedure is able, owing to the inclusion of the radius information, to generate larger perfusion regions from large feeding arteries, and vice-versa.

### Time discretization

Parameters $K_i$, $\beta _{i,j}$ and the compliance term $C_i$, computed with the data-driven approach described in Sects. 2.2-2.3, introduce significant nonlinearities in the compliant multi-compartment Darcy model (4). To cope with this issue, we rely on a linearized version of (4) obtained with a first-order finite difference discretization for the time derivatives and a semi-implicit treatment for the unknowns. Given a function *v*(*t*), we introduce a partition of time domain based on discrete instants $t^n =n\Delta t, \, n=0,1,\ldots$, with $\Delta t$ being the time discretization parameter, and we denote the approximated quantity as $v^n \simeq v(t^n)$.

Accordingly, the time-discretized multi-compartment Darcy model reads:

16 $$\begin{aligned} \left\{ \begin{array}{ll} \displaystyle - \nabla \cdot (K^n_i \nabla p^{n+1}_i) + (nL)_i C^n_i \frac{p^{n+1}_i - p^n_i}{\Delta t} + \sum _{j=1}^{3} \beta ^n_{i,j}(p^{n+1}_i - p^{n+1}_j) = &{}\\ \displaystyle \qquad \qquad = \theta _i + (nL)_i C^n_i \left( \frac{P_{\textrm{im}}(t^{n+1}) - P_{\textrm{im}}(t^n)}{\Delta t} \right) &{} \quad \text {in } \Omega ; \\ \displaystyle \frac{\partial p_i}{\partial {\varvec{n}}} = 0 &{} \quad \text {on } \partial \Omega , \\ \end{array} \right. \end{aligned}$$

where $P_{\textrm{im}}$ is the given intramyocardial pressure.

### Geometry reconstruction, numerical solution and simulation setup

Coronary blood flow (CBF) and perfusion simulations in hyperemic conditions (CBF-Perfusion simulations) performed on two patients from Centro Cardiologico Monzino in Milan. These patients are chosen among subjects with the following characteristics:

1. No history of previous major cardiac adverse events;
2. Symptoms of coronary artery disease including *angina pectoris*;
3. No actual anatomical signs of coronary artery disease (ruled out by imaging examinations), including coronary stenoses and atherosclerotic plaques.

These patients are therefore representative of a population of high-risk, symptomatic subjects that, however, have shown no sign of obstructive CAD or inducible myocardial ischemia.

For both patients, the left ventricular and coronary geometries are segmented from contrast-enhanced coronary computed tomographic angiography (cCTA) images, under the supervision of expert cardiologists. Coronary epicardial trees are segmented through a semi-automated procedure based on the colliding fronts algorithm in the VMTK software suite (Antiga et al. 2008): For each branch, lower and upper thresholds are chosen based on the local gray levels, whereas, for the algorithm parameters, default values are used. The left ventricular free wall is segmented using the fully automated tool TotalSegmentator (Wasserthal et al. 2023). Since the main interest is on perfusion of the left ventricle, coronary branches of the right coronary artery (RCA) perfusing the right ventricle are pruned from the segmentation. Geometries are meshed using VMTK: An example of the computational domains obtained is reported in Fig. 4.

For the numerical managing of the coupled problem, after time discretization we rely on a loosely coupled scheme: Time-discretized Navier–Stokes (see Gregorio et al. (2021)) and Darcy (16) problems are solved sequentially following a fixed-point iterative strategy with relaxation, by exchanging at each iteration *s* the coupling conditions (14) and (13). The corresponding iterations are reported in Algorithm 1, where the index $n+1$ for the current time instant is omitted for clarity, $\varepsilon$ is a given tolerance and $\eta$ is the relaxation parameter. As a stopping criterion, we consider the normalized difference between consecutive iterations $diff_x = \frac{\Vert {x^{(s)}-x^{(s-1)}}\Vert }{\Vert {x^{(s)}}\Vert }$ for each unknown *x*; the norm has to be intended as $[H^1]^3$ for velocities and $L^2$ for pressures.

**Algorithm 1:**
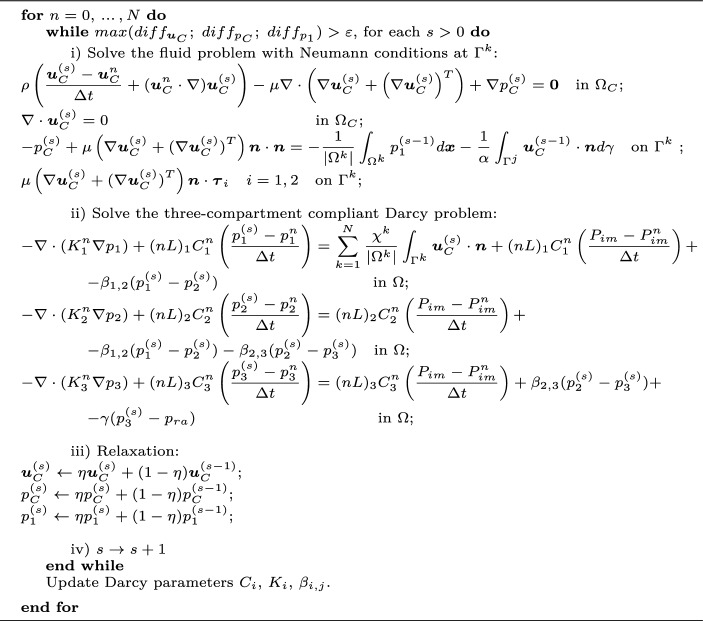
Solution of the time-discretized perfusion problem

Each subproblem is therefore solved independently with a separate solver. The fluid problem is solved using GMRES method with SIMPLE preconditioner (Deparis et al. 2014), whereas the compliant Darcy problem is solved using conjugate gradient method with a block Jacobi preconditioner.

Table 1 reports a list of the values for all the physical and numerical parameters used alongside an indication on how they are chosen. In particular, parameters $\gamma$ and $\delta _i$ are calibrated on a single patient (patient P1) with two targets:

1. Reproduce a distribution of pressure, along the microvasculature, matching experimental data from Chilian et al. (1989);
2. Reproduce an in-space average MBF, taken as the time-averaged capillary flow, matching the value obtained from the stress-CTP examination.

The same parameters are then used also for another patient P2, which represents, therefore, a validation case.

**Table 1:** List of parameters used for the simulations. Calibration refers to a trials-and-errors procedure on patient P1 to recover experimental data

Parameter	Value [compartments]	Source
Length density $(nL)_i$	[0.5 ; 15 ; 8000] mm mm$^{-3}$	Tomanek et al. (1991), Dedkov et al. (2006), Schwarz et al. (2020)
Specific permeability $\kappa$	$1.75 \times 10^{-10}$ m$^{-1}$	Michler et al. (2013), Papamanolis et al. (2021)
Coupling coefficient $\alpha$	$3 \times 10^{-10}$ m$^3$ s$^{-1}$ Pa$^{-1}$	Pelagi et al. (2024)
Morphometry factor $\delta _i$	[0.005 ; 0.05 ; 10] mm$^{-1}$ s$^{-1}$ Pa$^{-1}$	Calibration
Veins conductance $\gamma$	$8 \times 10^{-6}$ s$^{-1}$	Calibration
Right atrium pressure $p_{\text{ra}}$	2 mmHg	
Blood density $\rho$	1063 Kg m$^{-3}$	
Blood viscosity $\mu$	0.0035 Pa s	
Peak brachial pressure (P1)	140 mmHg	Measure
Heart rate at rest (P1)	63 bpm	Measure
Derived period T (P1, stress)	0.714 s	Pelagi et al. (2024)
Peak brachial pressure (P2)	130 mmHg	Measure
Heart rate at rest (P2)	55 bpm	Measure
Derived period T (P2, stress)	0.782 s	Pelagi et al. (2024)
Time discretization $\Delta t$	$2 \times 10^{-3}$ s	
Space discretization $h_{NS}$	0.4 mm	Sens. analysis
Space discretization $h_{Darcy}$	1.5 mm	Sens. analysis
Tolerance $\varepsilon$	$1 \times 10^{-10}$	
Relaxation factor $\eta$	0.1	Convergence test

All the simulations are run using the software $\texttt {life}^{\texttt {x}}$, a high performance library for Finite Elements simulations of multiphysics, multiscale and multidomain problems developed at MOX - Dipartimento di Matematica, in cooperation with LaBS - Dipartimento di Chimica, Materiali e Ingegneria Chimica, both at Politecnico di Milano (Africa 2022; Africa et al. 2024).

## Results

Simulations results are analyzed in Sect. 3.1 in terms of the following outputs:

1. Time evolution of the epicardial coronary flow, computed from the Navier–Stokes (NS) model, and of the venous outflow computed from the Darcy model;
2. Time evolution of the in-space average pressure within the Darcy compartments (intramural blood pressure);
3. Time evolution of arteriolar flow, capillary flow and vessel diameter at the subendocardium, mid-myocardium and subepicardium, computed from the Darcy model;
4. 3D distribution of capillary flow (from Darcy) and 3D velocity patterns in the epicardial arteries (from NS) along the whole heartbeat.

All the analyzed outputs are compared with in vivo human measurement or experimental data, when available. In Sect. 3.2, we report a comparison of the results with the outcomes of the model in the rigid case, i.e., vessels compliance is set to zero, whereas in Sect. 3.3 we report a sensitivity analysis with respect to the new parameters introduced with the proposed model.

### Analysis of hemodynamics results

**Fig. 5:**
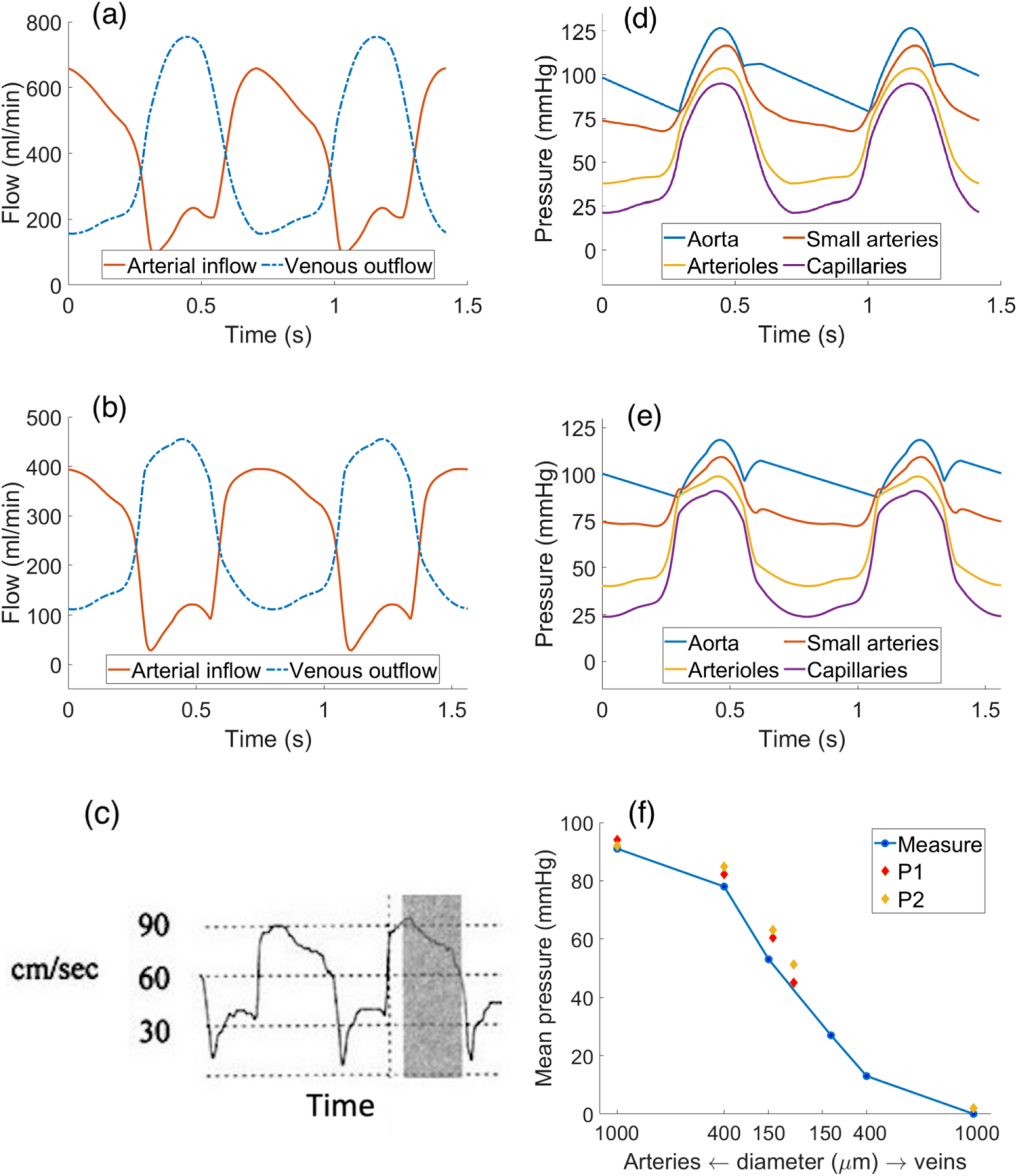
Left: total left arterial inflow and venous outflow over time for patient P1 (**a**) and P2 (**b**) as compared with a Doppler intracoronary velocity measure reproduced from Abe et al. (2000) (**c**). Right: average-in-space pressure in the Darcy compartment and aortic pressure over time for patient P1 (**d**) and P2 (**e**). **f** Averaged (both in space and in time) pressure values for both patients compared to experimental measures reported in Chilian et al. (1989). Straight blue lines only for visualization purposes

Figure 5a, b reports the time evolution of the arterial inflows and venous outflow (computed as the in-space average of $\theta _3$ as defined in (14)) for patients P1 and P2.

The physiological phasic pattern of high diastolic inflow and high systolic outflow can be clearly seen; moreover, we find an excellent accordance of arterial waveforms with in vivo experimental measures reported in Abe et al. (2000), Sunyecz et al. (2018) and depicted in Fig. 5c.

Table 2 summarizes some relevant quantities regarding the morphology of the flow waveform as compared with the in vivo Doppler measurements in rest conditions reported in Marcus et al. (1999). We report an excellent agreement in terms of the systolic/diastolic flow ratio in the left anterior descending (LAD) artery, whereas this ratio is underestimated in the case of the RCA. This is in agreement with our geometric model choices, which do not include the RCA branches perfusing the right ventricle, where the flow follows the aortic pressure waveform, thus featuring its peak during systole. Our computed peak/mean flow ratios show a slight underestimation compared to the measures, which could be due to small mismatches in the timing between the aortic and intramyocardial pressure waveforms used as input, resulting in a smoothed early diastolic flow peak.

Figure 5d, e reports the time evolution of the (in-space averaged) Darcy pressure for all the compartments, compared with the aortic pressure curve for patients P1 and P2. We can see that only small arteries follow the waveform of the aortic pressure, whereas arteriolar pressure is dominated by the intramyocardial pressure generated by contraction, with increasing pressure during diastole likely caused by the vessel filling with blood. The curves show blood pressurization due to contraction in systole. We report an in-time mean value of the Darcy pressures of 82.2, 60.4, and 45.0 mmHg (patient P1) and 84.9, 63.1 and 51.3 mmHg (patient P2) for the small arteries, arterioles and capillaries, respectively. All these findings reproduce what experimentally measured and reported in Chilian et al. (1989) and depicted in Fig. 5f. Notably, we do not observe any retrograde flow in the early systole, whose absence may be due to the hyperemic conditions.

**Table 2:** List of flow-related ratios computed by our model as compared to the in vivo Doppler measures reported in Marcus et al. (1999)

Ratio	P1: LAD-RCA	P2: LAD-RCA	Measure: LAD-RCA
Systolic/Diastolic peak flow ratio	0.35–0.38	0.31–0.31	0.37–0.97
Systolic/Diastolic mean flow ratio	0.35–0.39	0.28–0.29	0.22–0.85
Mean flow/Peak flow (Systole)	0.45–0.48	0.75–0.74	0.32–0.38
Mean flow/Peak flow (Diastole)	0.75–0.78	0.81–0.81	0.57–0.46

**Fig. 6:**
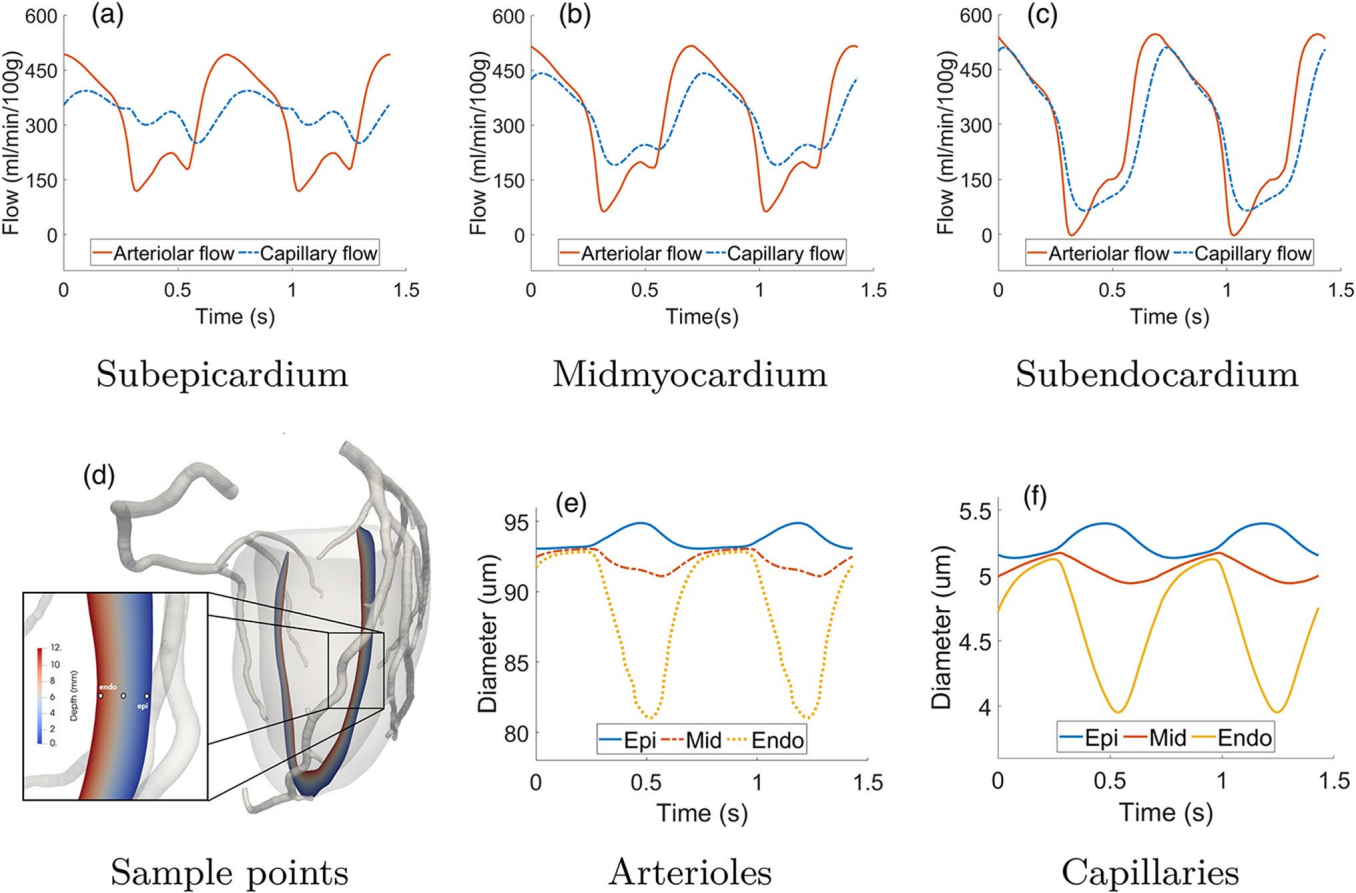
**a**–**c** Arteriolar and capillary flow over time at three sample points in the mid-anterior wall at different depth locations. **d** Localization of the three sample points used for the flow and diameter computation. **e**, **f** Arteriolar and capillary diameters over time at the same sample points

Figure 6a–c reports the arteriolar and capillary blood flow waveforms over time (patient P1), both computed at three sample points in the mid-anterior left ventricular wall located at different depths: subepicardium (1 mm below epicardial surface), mid-myocardium (in the middle), and subendocardium (1 mm below endocardial surface). The used sample points are depicted in Fig. 6d. For the computation of the arteriolar and capillary flows, we use the standard expressions:

17 $$\begin{aligned} {\left\{ \begin{array}{ll} {\text{Arteriolar flow rate}} = \beta _{1,2}(p_1 -p_2), \\ {\text{Capillary flow rate}} = \beta _{2,3}(p_2 -p_3). \end{array}\right. } \end{aligned}$$

From the waveforms of Fig. 6a–c, we can observe cyclic patterns of flow also in the microvasculature, similar to what obtained in the epicardial arteries (see Fig. 5a, b) with wider oscillations going from the epicardium to the endocardium. These oscillations are lower in the capillary rather than arteriolar flow, suggesting a dampening effect of the microcirculation similar to what is observed in the peripheral circulation as a response to the pulsatility of the aortic pressure. This effect is observed to an increasing extent from the subendocardium to the subepicardium, and it is also characterized by a time delay of the waveforms because of the compliance of the vessels wall.

Figure 6e, f reports the diameters of arterioles and capillaries computed from the pressures through relations (6)–(7) at the same sample points.

**Fig. 7:**
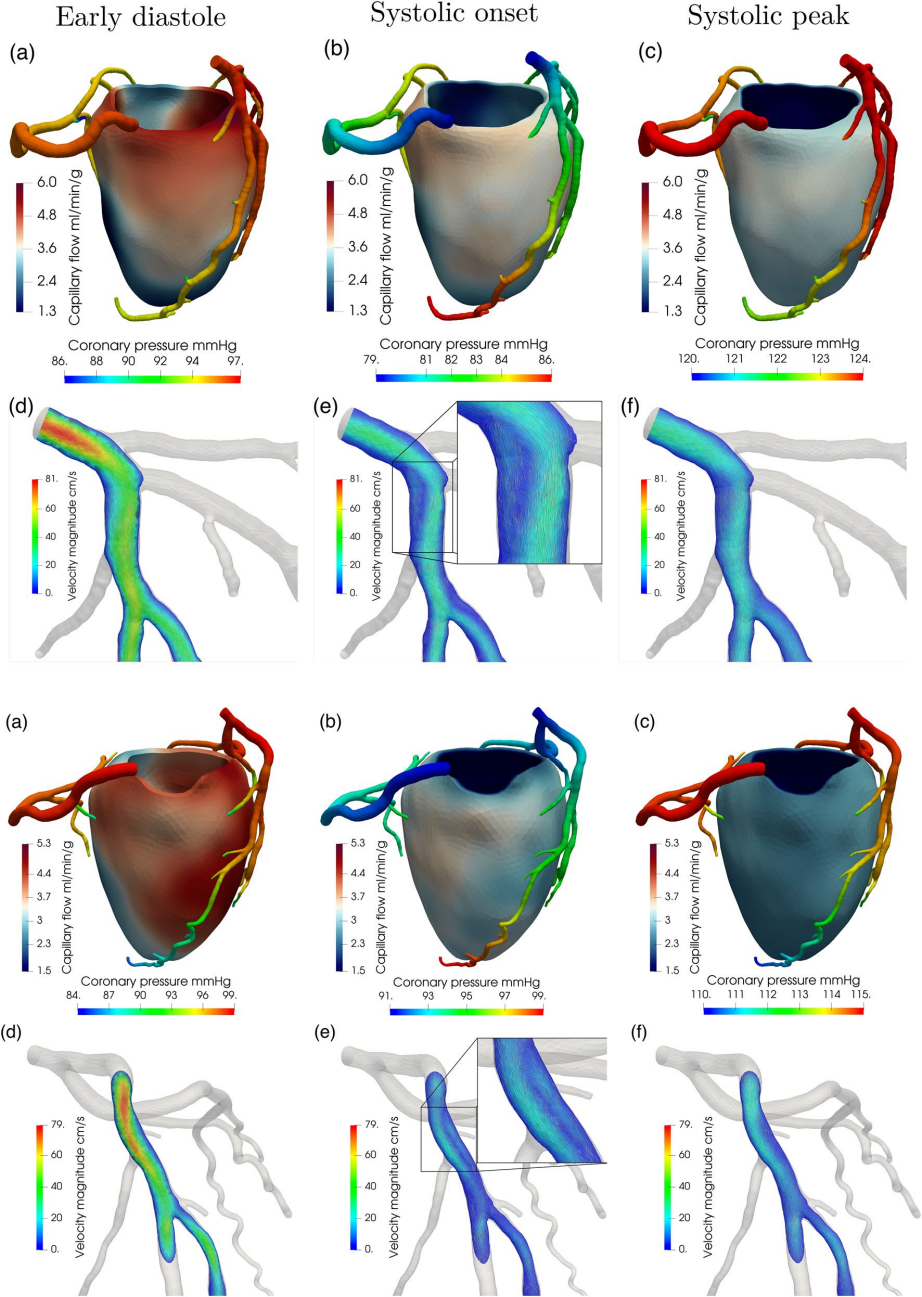
**a**–**c** 3D epicardial coronary pressure and capillary flow, computed as in the second line of 17, and **d**–**f** detail of the blood velocity in the left anterior descending artery, at three selected instants of the cardiac cycle. Notice that the scales for the coronary pressure are different at the three time instants to better highlight the key features. Patients P1 (top) and P2 (bottom)

Substantial differences are observed for both arterioles and capillaries at the various depths: slight increase in diameter at the subepicardium, slight decrease at the mid-myocardium and marked decrease at the subendocarium. This behavior, as well as the increasing flow oscillations, is consistent with the increase of the intramyocardial pressure from the epicardium to the endocardium and shows good agreement with experimental data of phasic diameter change in the arterioles (systolic/diastolic diameter change of $\simeq -20\%$ to $\simeq +2\%$ from endocardium to epicardium, as reported in Algranati et al. (2010) from various animal measures).

Figure 7 reports the 3D results for coronary flow, coronary pressure and capillary flow at three key time instants over the cardiac cycle in the two patients. Consistently with the flow waveforms reported in Figs. 5, 6, we observe that the diastolic flow is much higher than the systolic one both at the level of the capillaries and, in particular, of the epicardial arteries, despite the upstream pressure in the aorta being higher at the systolic peak. Also, we found a much higher pressure drop along the epicardial arteries at the early diastole ($\Delta p \simeq 9{-}15$ mmHg) rather than at systolic peak ($\Delta p \simeq 4{-}5$ mmHg), which is a consequence of the higher diastolic flow. At the systolic onset (Fig. 7b), the aortic pressure is at its lowest since the aortic valve is still closed; however, ventricular contraction is generating a high intramyocardial pressure which is the responsible of the inversion of the pressure gradient along the epicardial coronaries. This leads to a disturbed coronary flow (see representations in Fig. 7b) at the proximal bifurcations, featuring vortexes and regions of recirculation. However, this never results in a retrograde flow, most likely due to inertial effects and the brevity of this phase. Lastly, we observe that capillary flow distribution shows high regional heterogeneity in diastole, whereas it exhibits predominantly transmural heterogeneities in systole, when local hemodynamics is dominated by the intramyocardial pressure.

**Table 3:** Summary of hemodinamically relevant results computed for patients P1-P2. Velocities are averaged on the cross-section landmarks reported in Fig. 8, and diameters and flow rates are computed at the same landmarks

Artery	Quantity	P1	P2	Average
LAD	Cross-section avg. peak velocity cm s$^{-1}$	39	44	41.5 ± 1.5
	Flow rate ml min$^{-1}$	258	110	184 ± 74
	Diameter mm	5.4	3.4	4.4 ± 1
LCX	Cross-section avg. peak velocity cm s$^{-1}$	32	36	34 ± 2
	Flow rate ml min$^{-1}$	44	66	55 ± 11
	Diameter mm	3.0	3.6	3.3 ± 0.3
RCA	Cross-section avg. peak velocity cm s$^{-1}$	19	16	17.5 ± 2.5
	Flow rate ml min$^{-1}$	105	60	82.5 ± 22.5
	Diameter mm	4.8	4.1	4.45 ± 0.35

**Fig. 8:**
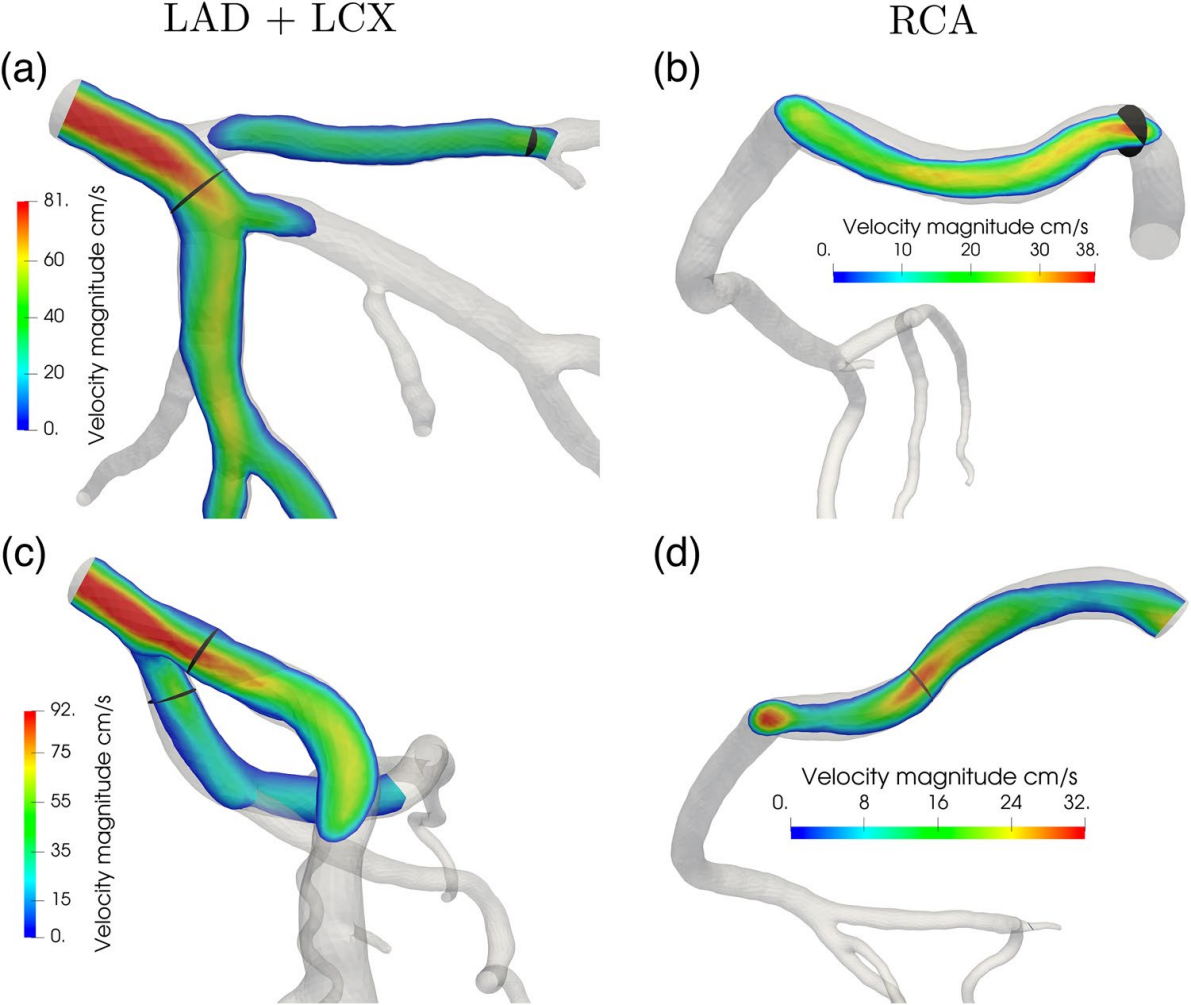
Velocity magnitude at the diastolic flow peak for the main artery branches: LAD, Left Circumflex artery, RCA for patient P1 (**a**, **b**) and P2 (**c**, **d**), with indication of the cross section used to find the peak velocity values. All the cross sections are placed in the most proximal segments of the corresponding artery, i.e., segment 1 for RCA, segment 6 for LAD and segment 11 for LCX

Figure 8 reports the blood velocity at diastolic flow peak computed in the three main arteries, whereas Table 3 reports a summary of the most hemodynamically relevant quantities obtained from the simulations, for both patients P1-P2. The obtained velocities in the LAD and LCX (left circumflex) arteries are in good agreement with the in vivo Doppler measures reported in Wieneke et al. (2005) for hyperemic conditions (48.8 ± 14.3 cm s^−1^ and $43.9 \pm 11.5 \, \text{cm} \, \text{s}^{-1}$ for the LAD and LCX). Compared to these measures, our velocity results are on the lower side due to our two cases showing no anatomical lesions in the coronary arteries, while the data reported in Wieneke et al. (2005) refer to a mixed population which includes also stenotic arteries where velocities may be much higher. In the case of the RCA, we observe an underestimation of blood velocity with respect to the measures ($42.4 \pm 12.4 \, \text{cm} \, \text{s}^{-1}$) which is due to the absence, in our model, of the blood flow perfusing the right ventricle.

Considering how inter-patient variability in the anatomy affects the hemodynamics, we find that the diameter of large coronaries has a substantial impact on the flow rate with negligible effect on blood velocity. Indeed, we see that the flow rate in a specific branch scales roughly with the cross-sectional area, whereas velocities tend to remain constant. We also observe a significant variability regarding the LAD-LCX flow subdivision, with values of 85–15% for patient P1 and 62–38% for patient P2, which is consistent with the LAD/LCX diameter ratio (1.80 and 0.944 for patient P1 and P2, respectively). This variability in flow subdivision can also be related to the myocardial mass perfused by each branch. Specifically, our patient P1 exhibits a high caliber first diagonal branch that originates from the proximal segment of the LAD and perfuses much of the territories normally perfused by the circumflex artery, which in this patient shows a much lower diameter. Conversely, in patient P2 the two branches have approximately the same caliber and a perfused myocardial mass much more balanced between them, resulting in a flow rate more evenly distributed. These results suggest that the specific anatomy plays a major role in flow subdivision and thus cannot be overlooked in computational frameworks that need an explicit prescription of such subdivision.

### Comparison with rigid microcirculation model

To highlight the importance of using a compliant formulation for the coronary microcirculation, we report here a comparison of the main model outcomes with respect to a scenario of rigid microvasculature.

**Fig. 9:**
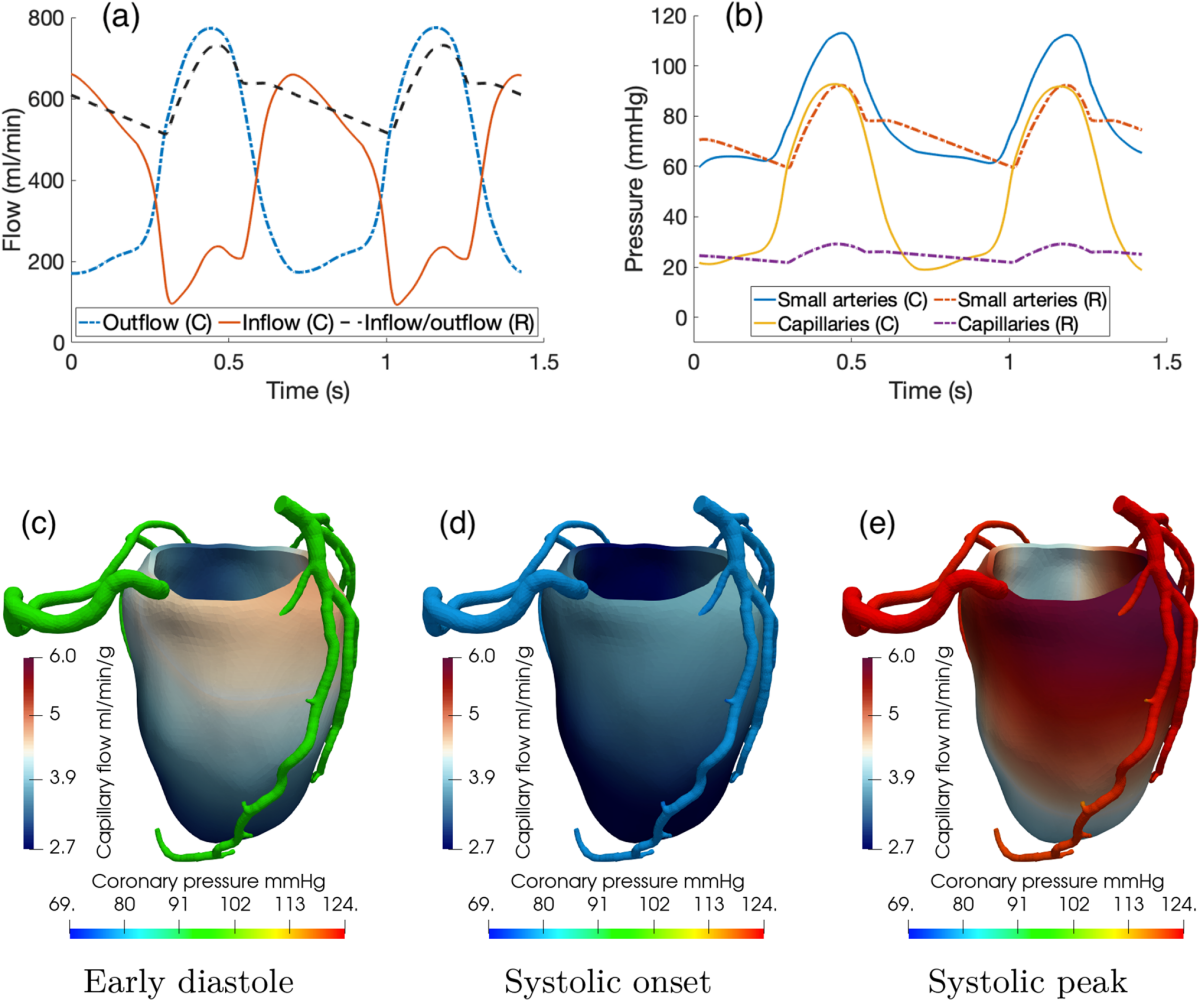
Comparison between arterial inflow and venous outflow flow rates (**a**) and pressures (**b**) over time for the compliant (**C**) and rigid (R) microcirculation models; arterial inflow and venous outflow are identical in case R. **c**–**e** 3D epicardial coronary pressure and capillary flow, computed as in (17), at three selected instants of the cardiac cycle. Simulation R for patient P1

In such scenario, vessels compliances $C_i$ in (4) are set to zero and vessels cross sections $A_i$ are constant in time, leading to constant Darcy parameters $K_i$, $\beta _{i,j}$ that we set according to our previous study (Pelagi et al. 2024). Figure 9 reports a comparison of the arterial inflow/venous outflow over time, as well as the 3D results of coronary pressure and capillary flow in the diastolic and systolic phases.

As we see from Fig. 9a, using a rigid model for the microcirculation results in an in-phase flow for the arterial and venous side of the coronary circulation, where the total arterial inflow is identical to the venous outflow. Unlike in the compliant case reported in Fig. 7, the systolic impediment effect is completely absent and the highest flows is obtained in systole. This can be seen also from the 3D results of Fig. 9e, where the capillary flow shows the highest values, along with the highest pressure drop in the epicardial coronaries, at the systolic peak. All of these outcomes, for the rigid model, are in contrast with the experimental observations and in vivo measures of coronary flow and pressure.

### Parameter sensitivity analysis

All the results presented in Sect. 3.1 are obtained using values for vessels length densities $(nL)_i$ (reported in Table 1) computed from literature morphometric studies. For the small arteries, the length density is obtained computing the total length of vessels with diameter $d > 100$ μm in the dataset from Schwarz et al. (2020) and dividing by the total volume of perfused myocardium reported in the same study. Values for the arteriolar and capillary length density are taken from Dedkov et al. (2006) and Tomanek et al. (1991), respectively, and adjusted to be representative of classes of vessels with a (albeit narrow) distribution of diameters rather than a single diameter value. Specific permeability $\kappa$ is computed through eq. (8) so that the resulting Darcy permeabilities $K_i$ are in line with previous computational studies (Michler et al. 2013; Papamanolis et al. 2021). Since inter-patient variability of these parameters could be relevant for predictive applications, we report here a sensitivity analysis performed to quantify how and to what extent these parameters , alongside the morphometry factors $\delta _i$, affect the results.

**Fig. 10:**
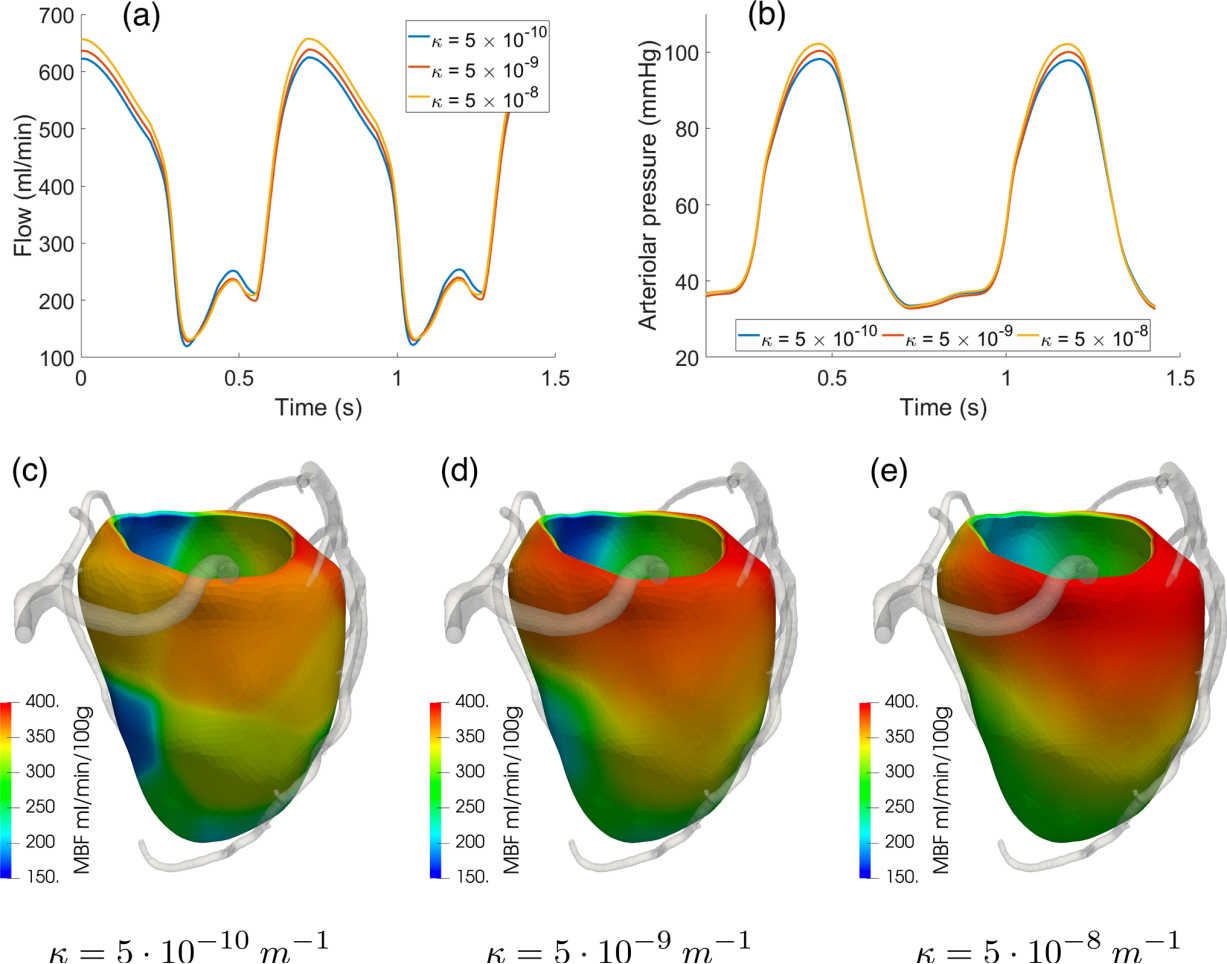
Effect of $\kappa$ in the range $(5 \times 10^{-10} {-} 5 \times 10^{-8}) \, \text{m}^{-1}$: total arterial inflow (**a**) and average-in-space arteriolar pressure (**b**) waveform over time. **c**–**e** 3D distribution of time-averaged capillary flow (MBF) over the myocardium

Figure 10 reports the results of the sensitivity analysis on the specific permeability $\kappa$ in the range $(5 \times 10^{-10} {-} 5 \times 10^{-8}) \, \text{m}^{-1}$. This affects the Darcy permeabilities $K_i$ (see (8)). For all the three values considered, we observe no relevant changes in the waveforms of arterial flow and in-space average arteriolar pressure over time (a-b) and in the overall time-averaged capillary flow (MBF, c-e). Regarding the latter quantity, we notice an increased heterogeneity in MBF regional distribution as $\kappa$ decreases. This can be interpreted in the following way: The mass source term in the first Darcy compartment ($\theta _1$ in (14)), representing blood flow in the different perfusion regions coming from the associated feeding arteries, is piecewise constant and shows discontinuities at the borders of the perfusion regions. However, these regions are not independent from each other, since spatial fluxes regulated by Darcy permeabilities $K_i$ lead to blood diffusion across the borders, smoothing the discontinuities resulting from the blood supply at the epicardial level. This smoothing effect among regions increases for higher values of $\kappa$. Physically, this means that $\kappa$ regulates how independent each perfusion territory is from the surrounding ones, with lower values associated with a more compartmentalized perfusion. Since the range of values analyzed is quite large (two orders of magnitude), we conclude that our model, in terms of in-space average quantities, has a rather low sensitivity toward $\kappa$.

**Fig. 11:**
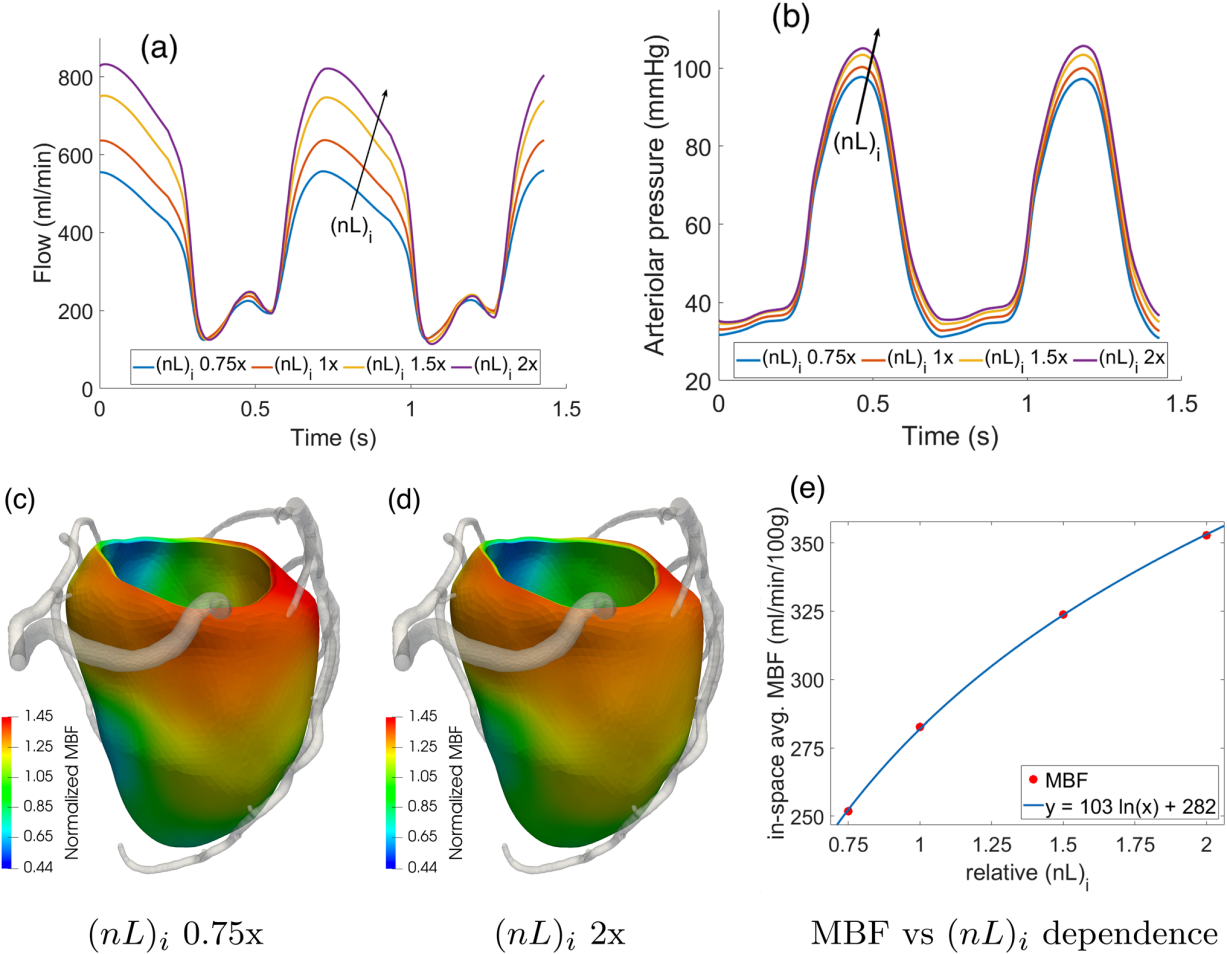
Effect of $(nL)_i$ values scaled homogeneously in the range 0.75x–2x w.r.t. the values reported in Table 1: waveforms over time of total arterial inflow (**a**) and arteriolar pressure (**b**). **c**, **d** 3D distribution of time-averaged capillary flow (MBF) normalized over the mean value to highlight the distribution; e) logarithmic fit built on the dependence of in-space averaged MBF vs the relative values of $(nL)_i$ w.r.t. the ones reported in Table 1

Figure 11 reports the results of the sensitivity analysis on the vessels length density $(nL)_i$ when the values associated to each Darcy compartment are scaled all by the same factor, in the range 0.75x–2x w.r.t. the values reported in Table 1. From the waveforms in Fig. 11a, b, we can see that the coronary flow and arteriolar pressure show a high sensitivity toward these parameters, which is not surprising given the direct proportionality between them and the inter-compartment Darcy conductances $\beta _{i,j}$ (see (11)). We notice also that the systolic flow is much less affected than the diastolic one, which is likely due to the fact that vessels length densities $(nL)_i$ affect also the compliance term in (4), leading to the balance of opposite contributions. Since diastolic flow is instead highly dependent on $(nL)_i$, we observe an overall logarithmic dependence of in-space average MBF vs $(nL)_i$, with the analytical fitted relationship reported in Fig. 11e. Other effects include higher arteriolar pressures (higher conductances in the Darcy model means that there is a higher pressure jump between the capillary compartment and the veins) and negligible effects on MBF distribution, which is mostly regulated by Darcy permeabilities $K_i$. Even if $K_i$ are directly proportional to $(nL)_i$ (see (8)), the range analyzed for this parameter is too narrow, compared to the sensitivity we found for $\kappa$, to lead to relevant differences.

**Fig. 12:**
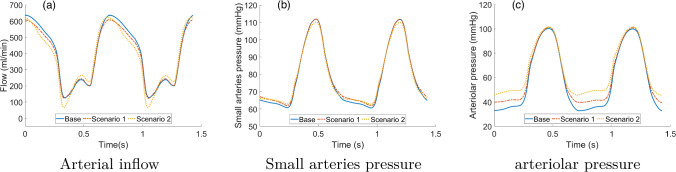
Effect of different combinations of $(nL)_i$ values on flow and pressure waveforms. The base scenario refers to the parameters reported in Table 1, whereas the other two scenarios represent relative scaling (as indicated) with respect to the base parameters

Since the model may show different responses to variations in single compartment length densities, for example due to the different constitutive relationships implemented for arterioles and capillaries, we also run simulations where $(nL)_i$ is changed non-uniformly in the three compartments. Figure 12 reports the results in terms of total arterial inflow and pressure in small arterioles and arterioles in the two following scenarios (base refers to Table 1),:

1. $(nL)_1$ doubled (w.r.t. base), $(nL)_2$ halved, $(nL)_3$ base,
2. $(nL)_1$ doubled, $(nL)_2$ base, $(nL)_3$ halved;

We can see that the flow waveform is predominantly affected by the capillary length density, with an increased oscillation in systolic flow and a slight decrease in diastolic flow in scenario 2. Pressure in the small arteries relatively unaffected in both scenarios, whereas diastolic arteriolar pressure shows a moderate ($\simeq 5$ mmHg) and relevant ($\simeq 10$ mmHg) increase in scenarios 1 and 2, respectively.

**Fig. 13:**
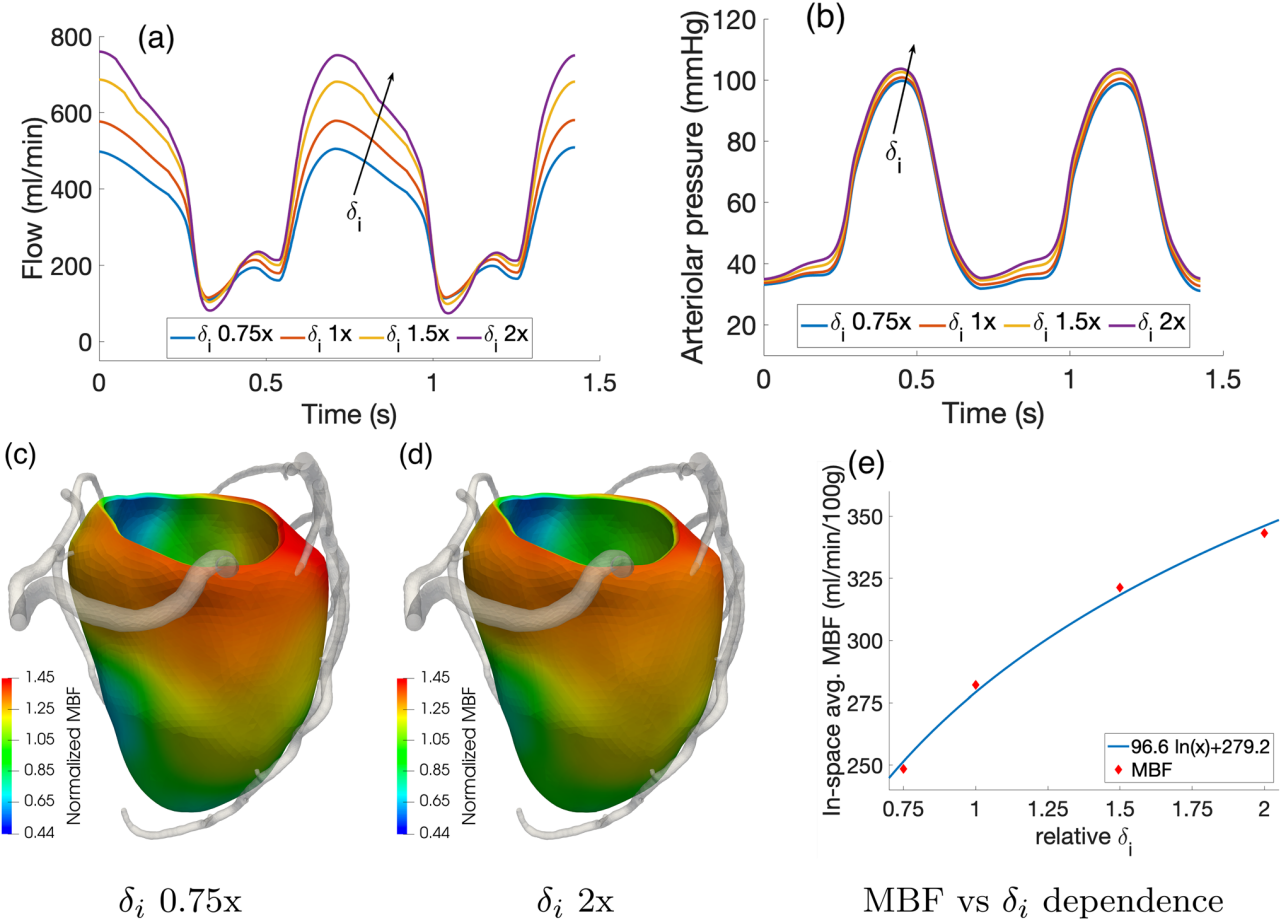
Effect of $\delta _i$ values scaled homogeneously in the range 0.75x–2x w.r.t. the values reported in Table 1: waveforms over time of total arterial inflow (**a**) and arteriolar pressure (**b**). **c**, **d** 3D distribution of time-averaged capillary flow (MBF) normalized over the mean value to highlight the distribution. **e** Logarithmic fit built on the dependence of in-space averaged MBF vs the relative values of $\delta _i$ w.r.t. the ones reported in Table 1

Figure 13 reports the results of the sensitivity analysis on the morphometry factors $\delta _i$ when the values associated to each Darcy compartment are scaled all by the same factor, in the range 0.75x–2x w.r.t. the values reported in Table 1. Similarly to what observed in the case of length densities, we report a high influence of $\delta _i$ on diastolic flow and overall in-space average MBF, but with negligible effect on MBF distribution.

From this analysis on the changes of the most relevant parameters, we can conclude that:

1. $\kappa$ mainly regulates the sharpness in the MBF gradients between adjacent perfusion regions. Higher values of $\kappa$ lead to smoother gradients and a more homogenized perfusion.
2. Parameters $(nL)_i$ regulate the pressure jumps between compartment *i* and its adjacents. We also observe that $(nL)_i$ has a relevant effect on diastolic flow, which greatly increases as $(nL)_i$ increase, while its effects on systolic flow is negligible. Out of all the Darcy compartments, the capillary length density $(nL)_3$ shows the highest impact on the results.
3. Parameters $\delta _i$ have an effect similar to $(nL)_i$, with a great increase of diastolic flow as $\delta _i$ increase.

## Discussion

Precise and effective modeling of coronary hemodynamics and cardiac perfusion is a daunting task due to the complexity of physical phenomena occurring during the heartbeat. The multiscale nature of the coronary circulation and the presence of a cyclic mechanical activation of the muscle represent key features that have a deep impact on the hemodynamics at the different scales. Several clinical studies demonstrated that the quantification of blood flow at the capillary level and over the various regions of the cardiac muscle adds significant prognostic value in the management of patients suffering from coronary artery disease (Pontone et al. 2019; Baggiano et al. 2020; Pelletier-Galarneau et al. 2019; do AH de Souza 2022). In these studies, in vivo functional imaging techniques such as nuclear imaging (positron emission tomography - PET, single photon emission computed tomography-SPECT) or stress-CTP are used to extract a 3D map of myocardial perfusion based on the radiotracer signal (in the nuclear imaging tests) or the time-attenuation curves of the contrast agent over the heartbeat. In all the cases, the myocardial blood flow is quantified using both diastolic and systolic flow values, so the development of models able to accurately capture both the diastolic and systolic 3D microcirculatory hemodynamics is of paramount importance.

In this work, we present a fully distributed, 3D mathematical model of coronary hemodynamics from the epicardial arteries (Navier–Stokes formulation) to the intramural vascular beds (multi-compartment Darcy formulation), that also includes for the first time the compliance of the microvessels and the presence of a cyclic external pressure representing the effects of cardiac contraction. The Darcy flow approach is a popular choice as it allows to describe microcirculation hemodynamics in a computationally efficient way without the need to solve 1D fluid dynamics equations in the full microvascular network, which is unfeasible due to its high computational cost (Chapelle et al. 2009; Papamanolis et al. 2021). Also, the multi-compartment formulation allows to take into account histological differences between intramural vessels of different diameter through vessel clustering into discrete classes (compartments) (Michler et al. 2013; Gregorio et al. 2021). As an alternative, there is also the possibility to couple Kirchhoff’s current law to discrete network (representing conservation of mass at the bifurcations) with Poiseuille law for the conductance of each segment, treated as a cylindrical element up to the arterioles (Schwarz et al. 2020): This approach has the advantage of being closer to the actual anatomy, considering a gradual reduction of the diameters as vessels progressively branch. However, the inclusion of the smallest arterioles and capillaries in such framework remains a challenge due to their huge number, and the assumption of Poiseuille law limits its applicability to steady flow conditions, neglecting cardiac contraction.

In our model, we include nonlinear constitutive relationships for microvessels compliance that we built on experimental data and we propose a new formulation for the Darcy parameters based on histologically relevant quantities, e.g., the local length density of vessels. This aspect is particularly relevant since such direct link can be exploited for a robust and precise calibration of the model, potentially tuning these parameters among the various myocardial regions, for example to distinguish between more and less vascularized territories.

Our model, applied to the simulation of hyperemic coronary flow in real clinical cases with geometries segmented from CT images, is successful in reproducing the phasic coronary flow pattern of high diastolic arterial inflow and systolic venous outflow, showing excellent agreement with experimental literature data with respect to the shape of the flow and pressure curves, the time evolution of diameters of microvessels and the differences at the various depths in the cardiac muscle.

As we show in Sect. 3.2, the use of a compliant model for the microcirculation is crucial to capture these features of the coronary circulation, since rigid models fail to correctly reproduce the physiology. This has also important consequences from a clinical standpoint, since an unphysiological systolic flow leads to the overestimation of the total myocardial perfusion and of the mean pressure loss along the epicardial coronaries. To account for the systolic impediment effect while still using a rigid microcirculation model, one may consider the use of an "effective" inflow boundary condition (as we did in Pelagi et al. (2024)) or on a modulation of the downstream vascular pressure (Zingaro et al. 2023), even though these choices are less representative of the actual physiology.

Finally, we notice that the calibration of the Darcy parameters seems to be robust with respect to the patients. Indeed, we use only limited information about P1 hemodynamics (namely the in-space average MBF) for the calibration and this leads to consistent results also for patient P2. For a more personalized setup, perfusion data from dynamic CTP acquisition at rest could also be used to calibrate the microcirculation parameters related to the topology of the vasculature, i.e., the length densities $(nL)_i$ and morphometry factors $\delta _i$ over the myocardium. In this setting, the constitutive relationships (5), (6), (7) (determining vessel area) would need to be replaced with suitable expressions representative of the rest state. The model can be then used to make predictions on perfusion relative to a virtual hyperemic scenario, using values for $(nL)_i$ and $\delta _i$ calibrated with rest perfusion data but with the constitutive relationships for $A_i$ relative to the hyperemic state, which incorporate the effects of maximal vasodilation. The biggest challenge for such framework is finding the correct expressions for $A_i$ at rest in patients affected by CAD. Indeed, in these cases, the autoregulation mechanisms are very likely to induce some degree of vasodilation in the microcirculation also at rest, to compensate the pressure loss due to the lesions in large vessels. Unlike in the hyperemic conditions, where maximal vasodilation is assumed for every patient, it can be difficult to assess the amount of vasodilation at rest, making the estimation of the $A_i$ curves a daunting task. Nonetheless, this approach holds great potential for fully personalized simulations, so addressing such challenges would be of high interest for future studies.

From a clinical perspective, our study provides an important contribution in view of the development of a tool to predict the whole hemodynamics in the coronary tree. To this regard, in Pelagi et al. (2024) we have shown that our model is successful in predicting fractional flow reserve (FFR), along the lines of previous works (Taylor et al. 2013; Tang et al. 2020; Pontone and Rabbat 2017; Ko et al. 2017; Lucca 2024), and in providing information on global Myocardial Blood Flow (MBF). Here, we instead focus on microcirculation, providing information on hemodynamics also at the tissue level. With this aim in mind, we remark that our model specifically adopts a fully distributed description of microvasculature hemodynamics and over the whole heartbeat, making it possible to quantify regional distribution of MBF over the myocardium. This will allow us in principle to include in our model the specific effects of other pathologies such as left ventricular hypertrophy and scenarios of altered contractility.

**Fig. 14:**
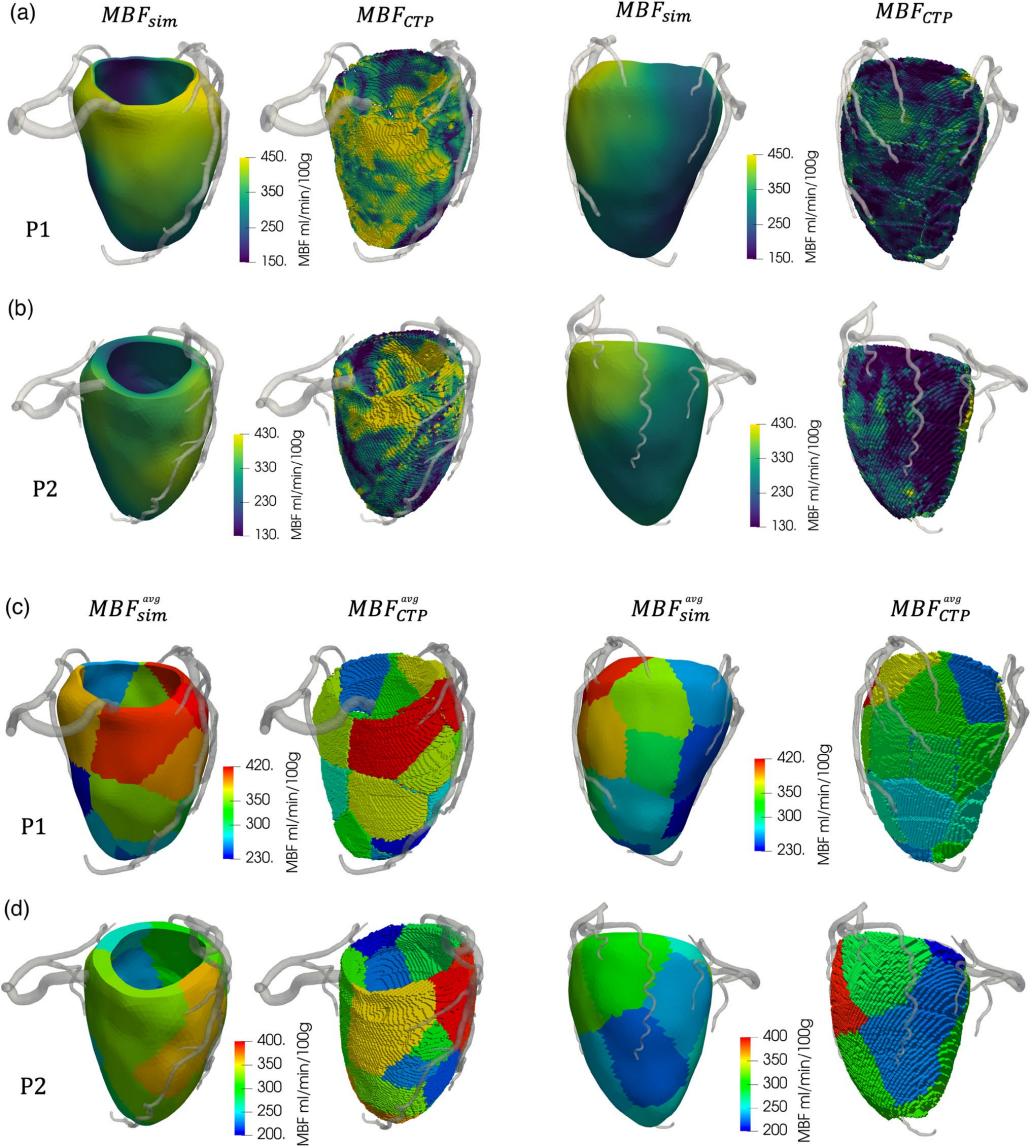
Comparison between simulated MBF (${\text{MBF}}_{\mathrm{sim}}$) and stress-CTP map (${\text{MBF}}_{\mathrm{CTP}}$) for patients P1 and P2. Left block: anterior view; right block: posterolateral view. **a**, **b** continuous MBF; **c**, **d** region-averaged MBF

In the direction of developing a tool which is able to predict also MBF distribution, we present here some further (preliminary) results on the comparison of our simulated MBF maps with those provided by the stress-CTP imaging acquisition, according to the protocol performed at Monzino Cardiology Centre. Figure 14 reports such comparison, where we report the continuous MBF over the whole myocardium and the MBF averaged over the single perfusion regions, both for patients P1 and P2. Notice that the computational domains are the same considered in the previous sections and reconstructed as described in Sect. 2.7.

From the results of Fig. 14a, b, we notice that perfusion maps from stress-CTP imaging show higher heterogeneities and sharper fluctuations in MBF if compared with simulated perfusion maps, which exhibit smoother and more homogeneous values. This can be due to the presence of some noise in the in vivo measurement, as well as to the limited number of perfusion regions that we adopt for myocardial subdivision. Considering the region-averaged MBF values in Fig. 14c and d, however, we can see that our model is able to reproduce the MBF distribution in both the analyzed patients, obtaining values in reasonable accordance with the clinical perfusion maps. Notably, we do not observe any region with ${\text{MBF}} < 150$ ml/min/100 g, which is the critical threshold for the detection of regions susceptible to inducible ischemia. This is in accordance with the outcomes of the stress-CTP examination, and thus we do not report any false-positive region in the two patients, despite the high heterogeneity in MBF values across the various regions.

When comparing numerical results with in vivo measures, particular attention needs to be paid to boundary conditions. To impose a hyperemic inflow conditions at the aortic root, we adapt the rest pressure measures following the methodology we proposed in Pelagi et al. (2024), where we validated our method by means of a comparison based on the hyperemic flow; thus, we believe that the comparison reported in Fig. 14 is meaningful. However, we are aware that a more fair comparison against hyperemic pressure measures will be mandatory for future studies. In any case, given that aortic pressure drives the total coronary flow but not how it is distributed across the different branches, our adaptation of the aortic pressure is expected to introduce an uncertainty which does not affect MBF spatial distribution. Regarding the imposed intramyocardial pressure $P_{\textrm{im}}$, instead, we compute it starting from the left ventricular pressure given by an electromechanics simulation in another (albeit anatomically accurate) ventricular geometry. Although we do not expect this choice to have an influence on the results in patients with a structurally normal heart (as the ones analyzed in this study), such influence may be significant in the case of structural abnormalities, e.g., left ventricular hypertrophy or myocardial fibrosis. To account for this, one may consider a modulation of the intramyocardial pressure across the different subdomains, based on subdomain structural characteristics such as volume, average wall thickness and presence of fibrotic tissue. Running an electromechanics simulation on the specific patient is another option, allowing to compute the active stress across the cardiac muscle in a personalized fashion and extract a continuous field for the intramyocardial pressure. The main disadvantage is the computational cost associated with this additional simulation.

From Fig. 14, we can see that, in some perfusion regions, there may be relevant discrepancies between ${\text{MBF}}^{\mathrm{avg}}_{\mathrm{comp}}$ and ${\text{MBF}}^{\mathrm{avg}}_{\mathrm{CTP}}$. Such differences may be due to different reasons, for example inaccuracies in the segmentation of the coronary tree and incorrect association between feeding arteries and myocardial mass resulting from the myocardial partitioning strategy we employ. In addition, we use constant values for the Darcy parameters all over the myocardium, overlooking the presence of heterogeneities in the myocardial properties. This heterogeneities are very likely related also to the specific anatomy of the epicardial tree, suggesting that a robust way to link anatomical features to microcirculation properties is a key aspect to include to correctly reproduce MBF distribution in a personalized way.

This work presents some limitations. Firstly, we are aware that two patients are not enough to conclude that our model can consistently predict the absence of perfusion defects in a given patient, i.e., assess the model specificity in a robust way. However, this is not a statistical study, rather a mechanistic one aimed at predicting hemodynamics, so that the number of patients is necessarily not high. Also, even though the analyzed subjects are symptomatic patients (as specified in Sect. 2.7), we do not examine any subjects with obstructive coronary artery disease, so we cannot assess the sensitivity of the model in the prediction of the presence and localization of perfusion defects. Further studies applied to a large and mixed population would be highly desirable to these aims. Still, we believe that the agreement we found in the MBF distribution between our simulations and the clinical maps (see Fig. 14) represents a very strong starting point in this direction.

Other limitations involve modeling choices and clinical applicability:

1. We do not include any branches of the RCA perfusing the right ventricle, which is motivated by the interest focusing on left ventricular perfusion. However, this likely impacts the hemodynamics in the proximal part of the RCA, leading to an underestimation of flow. Future studies should address this issue by considering right ventricular branches alongside appropriate outflow boundary conditions, such as calibrated Windkessel models.
2. We consider constant values along the whole vasculature (i.e., independent of the Darcy compartment) for both the viscosity $\mu$ and the specific permeability $\kappa$; see (8). These choices are motivated by noticing that both fluid-matrix interaction and rheology, although they may change along the vasculature, do not have an influence in recovering the right qualitative behaviors of the quantities of interest (phasic flow, systolic impediment, etc.). However, we believe that a deeper investigation of the quantitative influence of these parameters should be in order for future studies. For example, one may consider the influence of the Fåhraeus-Lindqvist effect on blood viscosity by modifying the expressions for permeabilities and conductances ((8) and (10), respectively), including a direct dependence on vessel diameter.
3. We do not consider transmural differences in the vessels properties. For example, it has been found that capillary density and diameter are higher at the subendocardium rather than at the subepicardium (Smith et al. 2014). Also, our model does not take into account that the intramural vessels (small arteries and microvessels) have a precise course into the cardiac muscle, that is a transmural course with increasing ramifications moving from the epicardium to the endocardium. Although we do not expect these features to have a relevant influence on phasic flow and regional distribution of MBF, they can substantially affect the transmural distribution of flow and should be taken into account for a more detailed study of blood perfusion across the myocardial wall. To this aim, one could consider a transmural modulation of vessels properties (length densities, morphometry factors), and a replacement of the permeability scalar fields $K_i$ with permeability tensors, featuring higher values in specific directions, such as the transmural one for small arteries or the one of the myofibers for capillaries. Since these directions change in space, this would require the definition of a moving reference frame in all points of the myocardium.
4. From a clinical standpoint, correct association between feeding arteries and perfusion territories is a key point. In this study, this is done generating perfusion regions starting from each coronary outlet at disposal. Given that the epicardial coronary trees we used are not limited to the main branches but include also smaller, transversal vessels (for example arising from diagonal and marginal branches) that become outlets in our segmentations, we consider this approach a reasonable strategy. However, according to morphometric data of a human coronary tree (Schwarz et al. 2020), many of the smaller penetrating vessels, branching from the main epicardial coronaries, may have a diameter lower than ${0.5\,\mathrm{\text {m}\text {m}}}$ and are likely to be missed in CT-based segmentations. The inclusion of such penetrating vessels may significantly affect the partitioning of the myocardium, leading to substantial differences in the association between myocardial mass and feeding arteries, thus representing a key point in the identification of coronary lesions responsible for perfusion defects. For these reasons, a more sophisticated partitioning strategy, including detailed morphometric data, will be an interesting topic for future development of this work.
5. For applications in clinical practice, an extended analysis with validation on a large and mixed population should be in order. This would require to strongly address the issue of the model calibration, potentially including space-dependent, personalized microcirculation properties. However, detailed microcirculation data can be acquired only through ev vivo experimental procedures, which obviously cannot be applied to living subjects. Also, the available data on human hearts are very limited and are representative of a specific anatomy, so they cannot be easily generalized to other subjects. Robust tools to couple the patient-specific anatomy of the epicardial arteries (visible from in vivo medical images) to specific characteristics of the downstream intramural vascular bed would be highly desirable. To this aim, the use of algorithms for the generation of synthetic vascular trees, informed by statistical distributions extracted from detailed topological data (Schwarz et al. 2020) and with a subsequent estimation of Darcy parameters from the generated networks, could represent valuable tools for a full personalization of the perfusion model.
6. Finally, for successful predictions of absolute MBF in patients with obstructive CAD, remodeling effects induced on microvasculature located downstream chronic occlusions and critical stenoses should be considered. These include arteriogenesis, representing enlargement of pre-existing collateral arteries, and angiogenesis representing sprouting of new capillary vessels. While angiogenesis can be modeled by increasing the length density of capillaries in the affected regions, collateral pathways could be, in principle, included through the source terms of the Darcy problem ($\theta _1$ in the first compartment, see (14)). Specifically, regions located downstream an occlusion could receive a fraction of the outflow of the nearby, healthy epicardial vessels. The identification of the determinants of these mechanisms, i.e., which regions benefit from them and their quantification, remains an open issue. Still, the distributed perfusion model we propose in this work can be applied, in combination with perfusion data from stress-CTP, to patients affected by obstructive CAD with the aim of exploring and quantifying these phenomena.

## Abbreviations

CAD: Coronary artery disease
CBF: Coronary blood flow
cCTA: Coronary computed tomographic angiography
FFR: Fractional flow reserve
LAD: Left anterior descending
MBF: Myocardial blood flow
NS: Navier–Stokes
RCA: Right coronary artery
stress-CTP: Stress computed tomographic perfusion

## List of symbols

$p_i$: Pore pressure in Darcy compartment *i*
$K_i$: Permeability of Darcy compartment *i*
$\phi _i$: Fluid volume fraction of Darcy compartment *i*
$\beta _{i,j}$: Inter-compartment conductance between Darcy compartments *i* and *j*
$P_\textrm{LV}$: Pressure in the left ventricular chamber
$$P_{\textrm{im}}$$: Intramyocardial pressure
$\theta _i$: Mass source/sink term in Darcy compartment *i*
$\gamma$: Conductance of venous circulation
$p_{\text{ra}}$: Right atrium pressure
$(nL)_i$: Vessels length density of Darcy compartment *i*
$A_i$: Average cross section of vessels in Darcy compartment *i*
$C_i$: Compliance of vessels in Darcy compartment *i*
$\mu$: Blood dynamic viscosity
$\kappa$: Specific permeability of cardiac tissue to blood flow
$\delta _i$: Morphometry conductance factor of Darcy compartment *i*
